# Evolution and Diversity of the Ras Superfamily of Small GTPases in Prokaryotes

**DOI:** 10.1093/gbe/evu264

**Published:** 2014-12-04

**Authors:** Kristin Wuichet, Lotte Søgaard-Andersen

**Affiliations:** Department of Ecophysiology, Max Planck Institute for Terrestrial Microbiology, Marburg, Germany

**Keywords:** small GTPase, evolution, GTPase-activating protein, Ras, MglA, signal transduction

## Abstract

The Ras superfamily of small GTPases are single domain nucleotide-dependent molecular switches that act as highly tuned regulators of complex signal transduction pathways. Originally identified in eukaryotes for their roles in fundamental cellular processes including proliferation, motility, polarity, nuclear transport, and vesicle transport, recent studies have revealed that single domain GTPases also control complex functions such as cell polarity, motility, predation, development and antibiotic resistance in bacteria. Here, we used a computational genomics approach to understand the abundance, diversity, and evolution of small GTPases in prokaryotes. We collected 520 small GTPase sequences present in 17% of 1,611 prokaryotic genomes analyzed that cover diverse lineages. We identified two discrete families of small GTPases in prokaryotes that show evidence of three distinct catalytic mechanisms. The MglA family includes MglA homologs, which are typically associated with the MglB GTPase activating protein, whereas members of the Rup (Ras superfamily GTPase of unknown function in prokaryotes) family are not predicted to interact with MglB homologs. System classification and genome context analyses support the involvement of small GTPases in diverse prokaryotic signal transduction pathways including two component systems, laying the foundation for future experimental characterization of these proteins. Phylogenetic analysis of prokaryotic and eukaryotic GTPases supports that the last universal common ancestor contained ancestral MglA and Rup family members. We propose that the MglA family was lost from the ancestral eukaryote and that the Ras superfamily members in extant eukaryotes are the result of vertical and horizontal gene transfer events of ancestral Rup GTPases.

## Introduction

In all organisms, GTP-binding proteins function to regulate a wide variety of cellular functions (Vetter and Wittinghofer 2001; Leipe et al. 2002; Wittinghofer and Vetter 2011). In eukaryotes as well as in prokaryotes, large GTP-binding proteins that consist of one or more domains in addition to the GTP-binding G domain are involved in ribosome biogenesis, tRNA modification, translation, and protein secretion. Eukaryotes also contain heterotrimeric G proteins consisting of the α-, β-, and γ-subunits and where the α-subunit contains the G domain in addition to the alpha helical domain. These proteins function together with G protein-coupled receptors in signal transduction. Moreover, eukaryotes contain small G proteins that only consist of the G domain and are referred to as the Ras superfamily of small GTPases. These proteins have well-characterized functions in regulation of cell polarity, motility, signal transduction, nucleocytoplasmic transport, and vesicular trafficking. Although heterotrimeric G proteins have not been identified in prokaryotes, recent experimental studies have provided evidence that small GTPases of the Ras superfamily are emerging as key players in the regulation of important functions including motility, cell polarity, predation, development, and antibiotic resistance in these organisms.

Ras superfamily GTPases are single domain nucleotide-dependent molecular switches of the TRAFAC class of P-loop NTPases (Leipe et al. 2002) and they share in common the G domain (Wittinghofer and Vetter 2011). As such they exist stably as either a GDP-bound form, which typically represents the inactive form, or a GTP-bound form that typically represents the active form and interacts with downstream effectors to elicit a response. The G domain contains four to five highly conserved sequence motifs (G1−G5) that are important for nucleotide binding, nucleotide-dependent conformational changes, and GTP hydrolysis. The conversion from the inactive GDP-bound form to the active GTP-bound form is stimulated by guanine nucleotide exchange factors (GEFs) (Bos et al. 2007). The conversion of the active to the inactive form is stimulated by GTPase activating proteins (GAPs) that stimulate the low intrinsic rate of GTP hydrolysis of the G domain (Bos et al. 2007). GAPs accelerate GTP hydrolysis by providing catalytic residues directly into the active site, correctly positioning catalytic residues intrinsic to the GTPases, or a combination of both (Seewald et al. 2002; Daumke et al. 2004; Pan et al. 2006; Scrima et al. 2008; Anand et al. 2013). In general, the primary residues for GTP hydrolysis by small Ras GTPases are an intrinsic glutamine residue in the GTPase and an arginine residue, referred to as the extrinsic arginine finger, provided by the GAP (Bos et al. 2007). Small Ras superfamily GTPases in eukaryotes are divided into at least five subfamilies (Jekely 2003; Wennerberg et al. 2005; Rojas et al. 2012), and the GEFs and GAPs for each subfamily are nonhomologous (Bos et al. 2007).

Over 20 years ago, MglA became the first identified bacterial member of the Ras superfamily of small GTPases (Hartzell and Kaiser 1991). Subsequent bioinformatics studies identified small Ras superfamily GTPases in diverse prokaryotes, but they were considered rare in comparison to their ubiquity in eukaryotes (Koonin and Aravind 2000; Leipe et al. 2002; Pandit and Srinivasan 2003; Dong et al. 2007). MglA, which is the best characterized small GTPase in prokaryotes, regulates motility and cell polarity in the δ-Proteobacterium *Myxococcus xanthus*. MglA and its cognate GAP, MglB, are dynamically localized in the rod-shaped *M*yx*. xanthus* cells such that MglA is at the leading cell pole and MglB is at the lagging cell pole and during cellular reversals the two proteins switch poles (Leonardy et al. 2010; Zhang et al. 2010). Furthermore, MglA interfaces with classic prokaryotic signal transduction components: The RomR response regulator and the Frz chemosensory system (Leonardy et al. 2007; Keilberg et al. 2012; Zhang et al. 2012) to regulate motility. Sequence and structural analysis of MglB revealed that it is a member of the roadblock/LC7 superfamily of proteins (Miertzschke et al. 2011), which is an ancient and widespread superfamily predicted to be involved in the regulation of NTPases (Koonin and Aravind 2000). This superfamily includes eukaryotic members of the dynein motor complex including the roadblock protein of *Drosophila melanogaster* that is involved in axonal transport and mitosis and the light chains of the flagellar motor (Bowman et al. 1999; Koonin and Aravind 2000). *Myx. xanthus* also employs a second small Ras superfamily GTPase in order to regulate motility, SofG, which is involved in correctly positioning the PilB and PilT ATPases that stimulate the extension and retraction of type IV pili for motility (Bulyha et al. 2013). Biochemistry and crystallography studies of the MglA and MglB homologs of *Thermus thermophilus* revealed that dimeric MglB interacts with monomeric MglA-GTP and that MglB stimulates GTP hydrolysis by MglA by correctly positioning the intrinsic catalytic glutamine and an intrinsic arginine finger of MglA at the active site (Miertzschke et al. 2011). SofG is a homolog of MglA that likely uses a similar catalytic mechanism given that it has a conserved arginine at the same position as in MglA and this residue is important for GTPase activity in vitro; however, MglB does not aid SofG GTP hydrolysis, and a cognate GAP protein has yet to be identified (Bulyha et al. 2013). Recently, the MglA homolog of the δ-Proteobacterium *Bdellovibrio bacteriovorus* was shown to interact with a RomR homolog as part of a system that is important for prey-invasion and type IV pili formation (Milner et al. 2014).

Small Ras superfamily GTPases have also been characterized in other bacteria, revealing additional links to two component signal transduction systems despite diverse outputs. Genome analysis of the Actinobacteria *Streptomyces coelicolor* showed that it encodes 13 copies of a highly conserved gene locus (conservon) (Bentley et al. 2002). Experimental and sequence analysis of one of the conservons revealed that it is composed of four proteins: A small Ras superfamily GTPase (CvnD9), a histidine kinase, a roadblock/LC7 family protein that is homologous to MglB (CvnB9), and a conserved protein of unknown function (Komatsu et al. 2006). Furthermore, the various interactions between these proteins are dependent on the catalytic activities of the kinase and the GTPase (Komatsu et al. 2006). Two of the conservons in *Streptomyces* species have been shown to play roles in the regulation of aerial mycelia formation, a complex developmental process that is initiated in response to nutrient limitation (Komatsu et al. 2003; Takano et al. 2011). In contrast, the lone conservon of the fellow Actinobacteria member *Mycobacterium smegmatis* has been shown to be involved in antibiotic resistance through its regulation of DNA gyrase (Tao et al. 2013). The study found that the Mycobacterium fluoroquinoline resistance protein A (MfpA) encoded immediately following the conservon interacts with the GTP bound form of the conservon GTPase (MfpB) and this interaction influences the interaction between MfpA and DNA gyrase (Tao et al. 2013).

Given the emerging importance of small Ras superfamily GTPases in prokaryotes, it is likely that future studies of these proteins will expand on their involvement in diverse fundamental cellular processes. Previous computational analyses provided glimpses of the diversity and evolutionary history of small GTPases. Here we chose to revisit a computational approach to the study of these proteins based on the rapid expansion of genomic data, some of which has come from underrepresented prokaryotic lineages that could expand upon our current knowledge. We performed a comprehensive phylogenomic analysis at a large scale using a set of 1,611 completely sequenced prokaryotic genomes from which we identified all small Ras superfamily GTPases and further explored them using sequence, genome context, and phylogenetic analyses. We identified two distinct subfamilies of small prokaryotic GTPases. Members of the MglA family are encoded by genes that are predominantly coupled (i.e., encoded near each other on the chromosome) to homologs of *mglB*, unlike members of the Rup (Ras superfamily GTPase of unknown function in prokaryotes) family. Both small GTPase families are distributed among wide-ranging and ancient taxonomic lineages, and sequence analysis of these proteins reveals three distinct types of catalytic mechanisms. Genome context and phylogenetic analyses allowed us to further distinguish specific groups within each family. Based on the extensive data obtained from these analyses in addition to phylogenetic analyses that included eukaryotic Ras superfamily members, we propose a scenario describing the evolutionary history that led to their distribution in extant organisms, starting with the emergence of the two ancestors of the MglA and Rup families in the last universal common ancestor (LUCA). These results also support the role of small Ras superfamily GTPases in diverse signal transduction systems and identify putative interaction partners, laying the foundation for their continued experimental characterization in the emerging field of prokaryotic small Ras superfamily GTPases.

## Materials and Methods

### Genome Set

All complete prokaryotic genomes 1,609 were downloaded from the National Center for Biotechnology Information (NCBI) Refseq (Pruitt et al. 2007) database on April 4, 2012. Due to our specific interest in members of Myxococcales, we also included the complete genomes of *Stigmatella aurantiaca* (Huntley et al. 2011) and *Corallococcus coralloides* (Huntley et al. 2012) from GenBank (Benson et al. 2007) as they were not yet available in Refseq at the time of genome collection.

### Software and Settings

The following software packages were used with the described settings unless otherwise specified. The HMMER3 software package (Finn et al. 2010) was used in conjunction with the Pfam26 domain library (Punta et al. 2012) for domain architecture analysis with default gathering thresholds. In the event of domain overlaps, the highest scoring domain model was chosen for the final domain architecture. We used BLASTP from the BLAST+ software package version 2.2.26 (Camacho et al. 2009) and considered hits with *e* values of 0.0001 or lower to be significant. Multiple sequence alignments were built using the l-ins-i algorithm of the MAFFT version 6.864b software package (Katoh et al. 2005). Sequence logos were created with WebLogo (Crooks et al. 2004). Phylogenetic trees were built using FastTree version 2.1.4 (Price et al. 2010), PhyML version 3.0 (Guindon and Gascuel 2003), and Phylip version 3.69 (Felsenstein 1989) (see following sections for tree construction details).

### Identification of MglB Homologs

The MglB sequence from *M*yx*. xanthus* (MXAN_1926) was used in a BLASTP query against the representative genome set and sequences with an *e* value of 0.0001 or lower were collected (63 sequences). The domain architecture of 58 of sequences consisted of a single Robl_LC7 domain without any overlapping domains and a total length ranging from 129–179 aa (supplementary table S1, Supplementary Material online). The remaining five sequences have an N-terminal Response_reg domain with MglB similarity confined to an uncharacterized C-terminal region. Domain architecture searches in Pfam confirm the existence of Response_reg and Robl_LC7 fusion proteins, and the BLASTP query suggests that a subset of Robl_LC7 family members do not match the current domain model to a significant degree.

From our data set, 650 sequences with a Robl_LC7 domain were identified in 230 genomes. In order to ensure that we did not overlook divergent MglB family members, the regions corresponding to the Robl_LC7 domain were used as BLASTP queries against the genome set, and all sequences with an *e* value of 0.0001 or lower were collected in order to identify potential homologs that did not meet the Pfam gathering threshold. Domain architecture analysis of the collected proteins confirmed that the regions of interest contain a Robl_LC7, MAPKK1_Int, or no characterized domains in the hit region. The MAPKK1_Int domain is part of the Profilin-like clan that also includes the Profilin and Robl_LC7 domains. This approach resulted in a final set of 749 MglB sequences from 238 genomes (supplementary tables S2 and S3, Supplementary Material online).

### Identification of Small GTPase Sequences

**Fig. 1.—:**
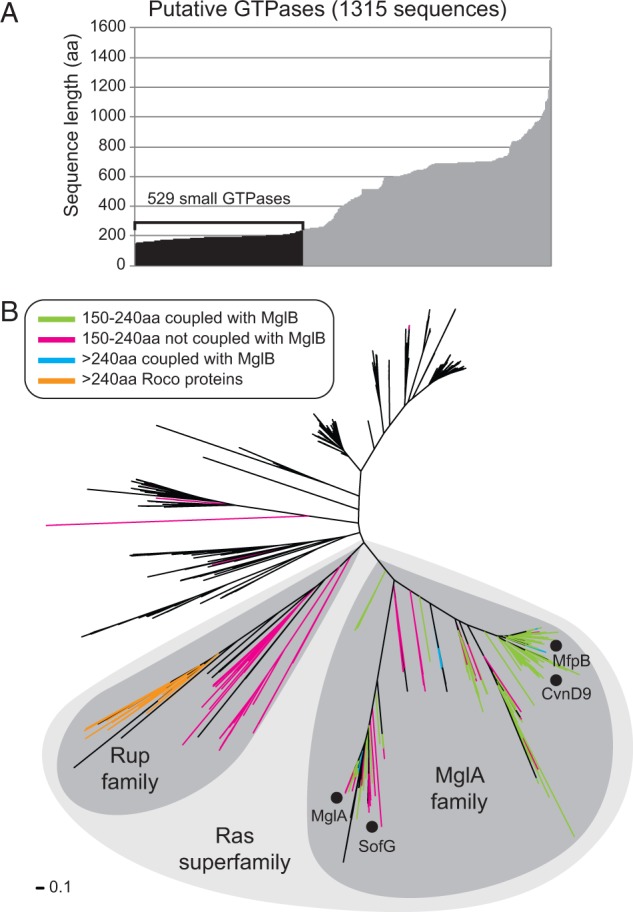
Identification of prokaryotic members of the Ras superfamily of small GTPases. (*A*) Sequence lengths of the 1,315 putative GTPases collected through domain architecture and BLASTP queries (Materials and Methods). The 529 small GTPases with sizes between 150 and 240 aa are indicated in black. (*B*) Phylogenetic tree built from a multiple sequence alignment of the 1,315 putative GTPases identified. Black dots indicate characterized members of the MglA family: MglA from *Myx. xanthus*, *T. thermus*, and *B. bacteriovorus*; SofG from *Myx. xanthus*; CvnD9 from *S. coelicolor*; and MfpB from *Myc. smegmatis*. Colors of branches indicate key features that allowed us to identify the MglA and Rup families. A GTPase is considered coupled with MglB if it is encoded within four genes of *mglB* in the genome. Sequences that contain N-terminal LRRs based on their match to the LRR domain model of Pfam are considered putative Roco proteins.

The MglA sequence from *M*yx*. xanthus* (MXAN_1925) was used in a BLASTP query against the genome set, and all sequences with an *e* value of 0.0001 or lower were collected. The 134 collected sequences revealed a variety of domain architectures; 15 sequences lacked any known domains, and the remaining sequences contained one of six domains (Arf, ATP_bind_1, GTP_EFTU, Gtr1_RagA, Miro, and Ras) from the P-loop_NTPase clan that contain 193 members. Four other domains of the clan were also found at significant levels, but were not present in the final architectures due to overlap with better scoring domains (supplementary table S4, Supplementary Material online). Based on the domain frequencies of the results (supplementary table S4, Supplementary Material online), the 639 sequences that contain Arf, ATP_bind_1, Miro, or Ras domains in their architecture were collected. In order to ensure that close homologs were not overlooked due to domain overlap, the regions corresponding to the four domains were extracted and used in BLASTP queries against our genome set. Sequences corresponding to the 2,164 hits with an *e* value of 0.0001 or lower were collected and used to build a multiple sequence alignment in MAFFT with default settings. A tree built from the alignment revealed a highly divergent subfamily of 830 putative ABC transporters (based on the presence of ABC_tran, ABC_tran_2, and/or AAA_21 domains). Although these domains are members of the P-loop_NTPase clan, the 830 sequences were excluded from further analysis to reduce noise in subsequent analyses. Their collection was due to four divergent ABC transporters that erroneously match the Miro domain, which were used in subsequent BLASTP queries, resulting in many ABC transporter hits. We also excluded 19 sequences shorter than 150 aa because all lacked one or more of the conserved regions associated with catalysis and nucleotide binding. A multiple sequence alignment of the remaining 1,315 sequences was built using MAFFT with default settings, and a phylogenetic tree was built from the region of the multiple sequence alignment corresponding to the G domain using FastTree. We found two distinct clades of small GTPases corresponding to Rup family members or MglA family members by comparing the tree topology with the domain architecture, sequence length, and genome context of the corresponding sequences (fig. 1). Sequences longer than 240 aa were excluded from further analysis resulting in the identification of 449 MglA sequences from 207 genomes and 71 Rup sequences from 43 genomes (supplementary table S2, Supplementary Material online).

*Schizosaccharomyces pombe* sequences from the Arf, Rab, Ran, Ras, Rho, Sar, and SRβ families of eukaryotic small GTPases described in previously analyses (Dong et al. 2007) were collected and used as BLASTP queries against the NCBI RefSeq database. Hits were filtered to include only eukaryotic sequences, and the best blast hits from *Arabidopsis thaliana, Caenorhabditis elegans, Chlamydomonas reinhardtii, Danio rerio, Dictyostelium discoideum, Drosophila melanogaster, Homo sapiens, Plasmodium falciparum, Rattus norvegicus, Ustilago maydis, Xenopus laevis,* and *Zea mays* were collected. Ras and Rho homologs were not collected from *A. thaliana, Ch. reinhardtii, P. falciparum,* and *Z. mays* because homologs were not within the top 100 hits.

### Phylogenetic Analysis of MglA, MglB, and Rup Homologs

**Fig. 2.—:**
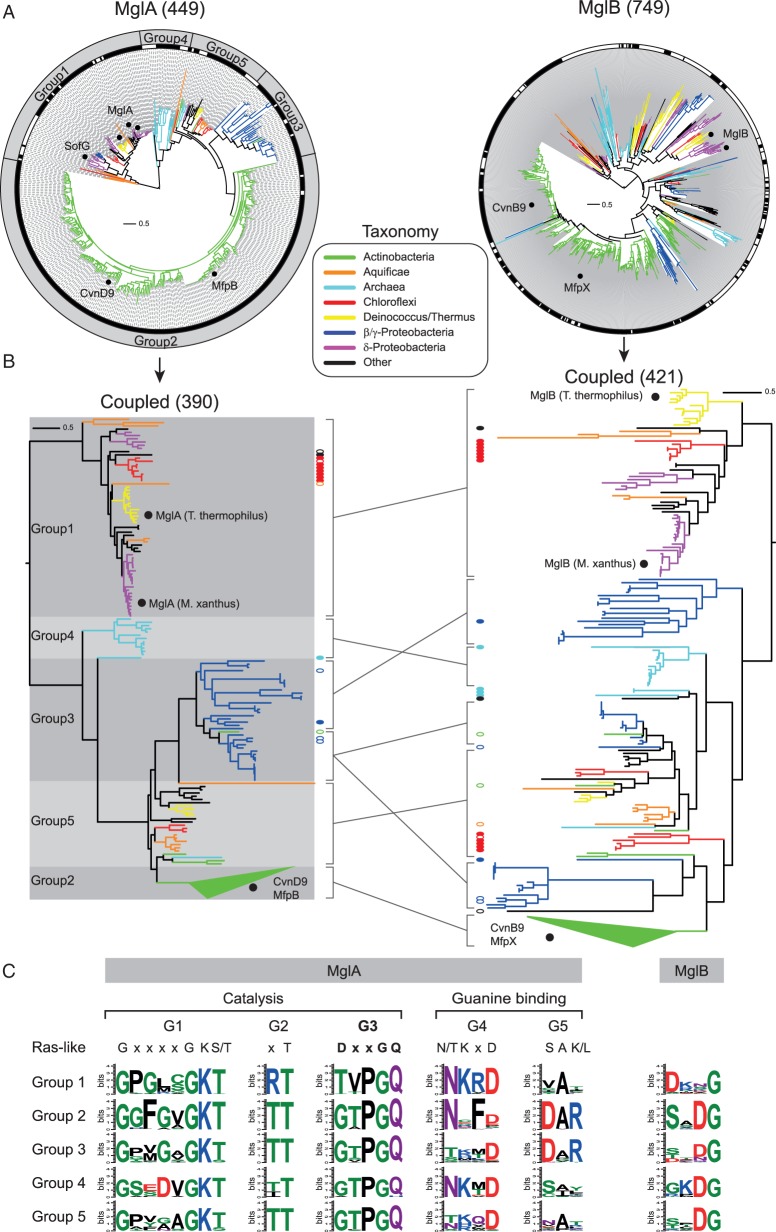
The MglA family contains five distinct groups. (*A*) Phylogenetic trees of MglA and MglB family members with branches colored based on taxonomy. Black marks in the rings around each tree indicate coupled sequences. The additional ring around the MglA tree identifies the five groups. Black dots on the MglA tree indicate characterized members: MglA from *Myx. xanthus*, *T. thermus*, and *B. bacteriovorus*; SofG from *Myx. xanthus*; CvnD9 from *S. coelicolor*; and MfpB from *Myc. smegmatis*. Black dots on the MglB tree indicate characterized members: MglB from *Myx. xanthus* and *T. thermus*; CvnB9 from *S. coelicolor*; MfpX is the MglB homolog coupled to MfpA in *Myc. smegmatis*. (*B*) Phylogenetic trees of coupled MglA and MglB sequences. Lines between the trees indicate coevolving groups. Tree branches and ovals are colored based on taxonomy. Black dots on the MglA tree indicate characterized members: MglA from *Myx. xanthus*, *T. thermus*; CvnD9 from *S. coelicolor*; and MfpB from *Myc. smegmatis*. Black dots on the MglB tree indicate characterized members: MglB from *Myx. xanthus* and *T. thermus*; CvnB9 from *S. coelicolor*; MfpX is the MglB homolog coupled to MfpA in *Myc. smegmatis*. Solid ovals in the MglA tree indicate an MglA sequence with two coupled MglB sequences that are similarly marked on the MglB tree, one of which is not found in the coevolving group of the MglB tree. Open ovals on the MglA tree indicate MglA sequences coupled to a single MglB that is similarly marked in the MglB tree, but the MglB is not found in the coevolving group of the MglB tree. The Group 2 MglA clade and corresponding clade of coupled MglB sequences are collapsed to aid visualization. (*C*) Sequence logos of selected regions of the multiple sequence alignments of the coupled MglA and MglB sequences. The G3 motif, which is used in the generation of the evolutionary scenario in figure 5*B*, is shown in bold.

MglA, MglB, and Rup homologs that were 150–240 aa were independently aligned using MAFFT, then the core region of the alignments corresponding to the following residue ranges were extracted for phylogenetic analyses: 14–189 of MglA from *M*yx*. xanthus* (MXAN_1925), 11–152 of a Ras-like sequence from *Mesorhizobium loti* (mll3243), and 17–125 of MglB from *M*yx*. xanthus* (MXAN_1926). A multiple alignment was also made of the collective set of small MglA and Rup homologs, which included eukaryotic homologs, the core region corresponding to residues 15–187 of MglA from *M*yx*. xanthus* (MXAN_1925) was extracted for phylogenetic analyses. In total, 1,000 bootstrapped replicates were made from each of the four core alignments using SeqBoot from Phylip with default settings. Trees were built from each replicate using FastTree with default settings and then a consensus tree was constructed with Consense from Phylip using default settings. Each consensus tree was used as a starting tree in PhyML in order to optimize the branch lengths using empirical frequencies and SPR (Subtree-Pruning-Regrafting) topology searches without altering the topology. Genome context and taxonomy data were used to identify conserved groups in the tree for classification (fig. 2 and supplementary table S2, Supplementary Material online).

## Results

### Identification of Prokaryotic Members of the Ras Superfamily of Small GTPases

Ras superfamily GTPases are composed of a single P-loop GTPase domain that typically has a length of 160–180 aa (Wittinghofer and Vetter 2011); however, P-loop GTPases can also be components of multidomain proteins involved in a variety of cellular processes. P-loop GTPases are represented within the P-loop_NTPase clan in Pfam that includes 193 domain models (Punta et al. 2012), and over 300,000 sequences in a set of 1,611 completely sequenced prokaryotic genomes available in April 2012, which comprehensively covers all major phyla, encode one or more of those domains. Nearly 7,000 of these sequences are 180 aa or less, making it computationally challenging to identify which of these small NTPases are members of the Ras superfamily. We, therefore, chose to identify prokaryotic Ras superfamily members by using a comprehensive approach that included sequence conservation, sequence length, phylogenetic, and domain architecture analyses. First, we performed a BLASTP search using MglA from *M*yx*. xanthus* as a query and found that the most significant hits matched ten Pfam domain models from the P-loop_NTPase clan. However, some significant BLASTP hits (*e* value less than 0.0001) did not significantly match any Pfam domain models despite the presence of conserved motifs associated with GTPases revealing that domain models alone are not sensitive enough to identify all small Ras GTPases in prokaryotes (supplementary table S4, Supplementary Material online). Therefore, we used BLASTP analysis in addition to Pfam models by first collecting the 639 sequences from our set of 1,611 genomes that match the four most prevalent domain models identified in the BLASTP analysis (supplementary table S4, Supplementary Material online, and Materials and Methods) and then extracted the regions corresponding to the G domain for use in additional BLASTP queries against our genome set. Newly identified sequences that were significant hits in these BLASTP analyses were collected and added to the previous set resulting in 1,334 sequences containing a G domain.

The collected GTPases range in length from 59 to 1,448 aa (fig. 1*A*). Because the Ras superfamily GTPases typically has a length of 160–180 aa we constructed a multiple sequence alignment of the 47 sequences shorter than 160 aa. This alignment revealed that all sequences shorter than 150 aa had lost one or more of the regions that are essential for nucleotide binding and/or hydrolysis. These 19 sequences are likely truncations that are the result of mutations or sequencing errors and were not considered further resulting in a set of 1,315 G domain containing proteins. MglA from *M*yx*. xanthus*, *T. thermus*,** and *B. bacteriovorus* is 195 aa, 196 aa, and 197 aa, respectively, CvnD9 from *S. coelicolor* is 176 aa, MfpB from *M*yc*. smegmatis* is 193 aa, and SofG is 239 aa (Bulyha et al. 2013). Therefore, we specifically identified those G domain containing proteins that have a size between 150 and 240 aa. Among our set of 1,315 sequences, 529 proteins fulfilled this criterion (fig. 1*A*).

Finally, we built a phylogenetic tree of the 1,315 collected sequences that are longer than 150 aa, and we then located the branches of the tree that correspond to sequences that have a size of 240 aa or less. Surprisingly, 520 of the 529 sequences that are 240 aa or less map to a large clade of 609 sequences that is composed of two distinct subclades (fig. 1*B*). Based on the cogent structure of the phylogenetic tree, we feel confident that our approach successfully identified all prokaryotic members of the Ras superfamily of small GTPases (table 1 and supplementary table S2, Supplementary Material online).

**Table 1:** Sequence Length and MglB Coupling of MglA and Rup Members

	MglA Subclade	Rup Subclade
All sequences	488	121
Small sequences (<240 aa)	449	71
Coupled to MglB	404	0
Small sequences coupled to MglB	390	0

### Identification of the MglA and Rup Families of the Ras Superfamily of Small GTPases

It has been found that genes encoding small GTPases and MglB homologs are often coupled in prokaryotic genomes (i.e., encoded near each other on the chromosome) (Koonin and Aravind 2000). To determine the co-occurrence of genes for small GTPases and MglB homologs, we set out to comprehensively identify MglB homologs in our set of 1,611 prokaryotic genomes in an iterative process. To this end, we first performed a BLASTP analysis using MglB from *M*yx*. xanthus* as a query sequence. MglB is a member of the Roadblock/LC7 family of proteins (Koonin and Aravind 2000; Miertzschke et al. 2011), and all but five of the significant hits (*e* value less than 0.0001) obtained in this search matched the Robl_LC7 domain model from Pfam that corresponds to this family (supplementary table S1, Supplementary Material online). The remaining sequences did not match any Pfam models demonstrating that as seen in the MglA BLASTP analysis the Robl_LC7 domain model alone is also not sensitive enough to identify all members of this family. Therefore, we used BLASTP analysis in addition to Pfam domain models by first collecting the 657 sequences in our genome set that significantly match the Robl_LC7 domain model, and then extracting the regions corresponding to the Robl_LC7 domains in these sequences for use as BLASTP queries against our genome set. Significant hits from these searches were added to the previous collection resulting in a total set of 749 MglB family members (supplementary tables S2 and S3, Supplementary Material online). Interestingly, 390 small GTPases are encoded within four genes of a gene encoding one of these MglB sequences and all these GTPases map to only one of the two small GTPase subclades in the GTPase tree. Because MglA from *M*yx*. xanthus*, *B. bacteriovorus*, and *T. thermophilus* maps to this subclade, we refer to this group of GTPases as the MglA family (fig. 1*B*).

The clade with small GTPases that are not coupled to MglB homologs includes a subfamily of longer sequences the majority of which contain N-terminal LRRs (leucine-rich repeats) in addition to the GTPase domain and a conserved C-terminal COR (C-terminal of Roc [Ras of complex proteins]) domain (fig. 1*B*). This domain architecture is indicative of Roco proteins, which contain the Roc GTPase domain (Bosgraaf and Van Haastert 2003; Gotthardt et al. 2008). Previous bioinformatics and experimental work showed that there is strong similarity between the Roc and Ras GTPase domains (Bosgraaf and Van Haastert 2003; Gotthardt et al. 2008). Given the grouping of the Roco proteins with the small GTPases that are not part of the MglA family, we henceforth refer to this novel family of small prokaryotic GTPases as the Rup (Ras superfamily GTPase of unknown function in prokaryotes) family (fig. 1*B*).

### Classification of the MglA Family

Among the 520 identified Ras superfamily GTPases that are 240 aa or less, 449 belong to the MglA family and were identified in 207 genomes (fig. 2*A*). Phylogenetic analysis of the MglA family members revealed five distinct groups based on tree topology and taxonomic distribution (fig. 2*A*). Subsequent genome context analyses that are described later additionally support the assignment of these groups. Although MglA family members are distributed among a variety of diverse genomes overall, members of Groups 2–4 are each confined to specific taxonomic groups unlike members of Groups 1 and 5 (fig. 2*A*). CvnD from *S. coelicolor* and MfpB from *M*yc*. smegmatis* are members of Group 2. We find that Group 2 members are encoded in 44% of the 169 surveyed Actinobacteria genomes including all sequenced genomes of the industrially important *Streptomyces* spp, pathogenic *Mycobacterium* spp, and the nitrogen-fixing plant symbionts *Frankia* spp (Ventura et al. 2007). Group 3 members are almost exclusively encoded in the genomes of β/γ-Proteobacteria including pathogens such as *Neisseria* spp., *Stenotrophomonas* spp*.*, and *Xanthomonas* spp. Interestingly, Group 4 members are almost exclusively present in methanogenic Euryarchaeota, and they are encoded in 56% of the surveyed genomes from this lineage. Both Group 1, which includes MglA and SofG of *M*yx*. xanthus* as well as MglA of *B. bacteriovorus* and *T. thermophilus*, and Group 5 members are found in Aquificae, Chlorobi, Chloroflexi, Deinococcus/Thermus, and δ-Proteobacteria genomes. We also identified Group 1 members in Acidobacteria, Deferribacteres, Dictyoglomi, Fibrobacteres, Gemmatimonadetes, β/γ-Proteobacteria, and Thermodesulfobacteria lineages, whereas Group 5 members are additionally encoded in Archaea, Actinobacteria, Cyanobacteria, and Verrucomicrobia genomes. Notably, MglA family members are present in many ancient, deep-branching lineages of Bacteria as well as Archaea.

### Coevolution of MglA and MglB

As described, we identified 749 MglB homologs in 238 genomes. In total, 200 genomes encode both an MglA family member and an MglB family member. This large genomic overlap supports that we correctly identified MglB family members despite finding 33% more of them in comparison to MglA family members. Phylogenetic analysis of the 749 MglB family members resulted in a tree that did not have a topology matching that of the MglA tree (fig. 2*A*). To better understand the evolutionary relationship between MglA and MglB, we identified the branches of the MglA and MglB trees that correspond to coupled sequences (i.e., MglA and MglB sequences that are encoded within four genes of each other) whereas the remaining sequences are deemed “orphans” (fig. 2*A* and supplementary tables S2 and S3, Supplementary Material online). Among the 449 MglA family members, 390 are coupled to an MglB. Among the 749 MglB family members, 421 are coupled to an MglA. We found that the lack of a 1:1 relationship between these MglA and MglB sequences was due to a subset of systems that have two MglB sequences coupled to a single MglA sequence.

To determine whether coupled MglA and MglB sequences coevolved, we built phylogenetic trees from multiple sequence alignments of the coupled MglA and MglB sequences (fig. 2*A*). We found that the groups originally characterized in the MglA tree are readily identifiable in the MglB tree. Interestingly, Group 3 systems form a single clade in the MglA tree, but three distinct clades in the MglB tree (fig. 2*B*). One of these clades in the MglB tree is associated with systems that have only one coupled MglB, whereas the other two clades are associated with systems that have two coupled MglB members. This pattern suggests that the MglB sequences from 1:1 versus 1:2 MglA:MglB systems are under different evolutionary pressures that obscure their predicted common origin. Furthermore, a small subset of Group 1 MglA sequences are coupled to two MglB sequences, and in these systems, only one MglB is present in the coevolving clade. The remaining cases of 1:2 coupled MglA:MglB are due to duplicated conservons that are encoded adjacent to each other, one of which has lost the Group 2 *mglA* gene (supplementary table S2, Supplementary Material online). Despite these discrepancies, overall the data support that the coupled MglA and MglB proteins are members of coevolving GTPase and GAP systems.

So far, no orphan MglB sequences have been experimentally characterized. The orphan small GTPases SofG in *M*yx*. xanthus* and MglA in *B. bacteriovorus* are the only experimentally characterized orphan MglA family members. Lack of any of these proteins causes significant defects demonstrating that these orphan genes are functionally important. The above analyses identified 59 MglA homologs and 328 MglB homologs that are not coupled to an MglB or MglA homolog, respectively. Inspection of the MglA and MglB tree topologies supports that orphan sequences can arise from a variety of events (fig. 2*A*). For example, in the MglA tree there is a large clade of actinobacterial sequences comprising all of the Group 2 MglA members, and in the MglB tree there is a corresponding large clade of actinobacterial MglB sequences most of which are coupled to the Group 2 MglA members (fig. 2*A*). However, there is a patchy distribution of orphan MglB sequences within this clade, many of which are encoded by genes that are flanked by conservon genes, indicating gene loss of the Group 2 *mglA* in these systems. On the other hand, there is a second small clade of actinobacterial MglB family members that are exclusively orphans (fig. 2*A*) and the corresponding genes are not flanked by genes encoding conservon components. This pattern could be the result of an ancient duplication or horizontal transfer of an MglB gene. For the purposes of this study, we chose to focus on coevolving, coupled MglA and MglB sequences for further investigation because they include the majority of MglA proteins.

### MglA Catalytic Mechanism Diversity

P-loop GTPases utilize five conserved regions to bind nucleotides and carry out GTP hydrolysis: The G1, G2, and G3 regions coordinate the β- and γ-phosphates of the nucleotide in addition to the essential magnesium cation for catalysis, whereas the G4 and G5 regions are responsible for guanine-binding specificity (Bourne et al. 1991; Leipe et al. 2002) (fig. 2*C*). The G1 region contains the P-loop (GxxxxGK[T/S]). The G2 region has a conserved threonine. The G3 region contains a conserved DxxG motif and members of the Ras superfamily of small GTPases have an additional conserved glutamine (DxxGQ) that coordinates the catalytic water for a nucleophilic attack of the γ-phosphate of GTP (Anand et al. 2013). The [NT]KxD motif of the G4 region is the primary determinant for the specificity of guanine over other bases, but the serine in the SA[KL] motif of the G5 region has also been shown to interact with the guanine base (Wittinghofer and Vetter 2011).

We analyzed members of each MglA group for the conservation of the G4 and G5 regions to investigate their ability to bind guanine nucleotides. The G4 region in Groups 1, 4, and 5 is consistent with the established consensus motif and the G5 region is poorly conserved (fig. 2*C*). On the other hand, the G4 nucleotide-binding motifs of Group 2 and Group 3 proteins deviate from the [NT]KxD motif (fig. 2*C*). Most notably, they often lack the conserved lysine involved in guanine base interaction. Experimental evidence clearly demonstrated that the Group 2 MglA CvnD9 has GTPase activity (Komatsu et al. 2006). We speculate that the conserved arginine in the unusual G5 motif (DAR) in these two groups may compensate for the absence of the lysine in the G4 motif given their similar physicochemical properties (fig. 2*C*). Furthermore, the strong conservation of a phenylalanine in the Group 2 G4 motif (NxFD) suggests that this is an important residue in these systems because this position is not typically well conserved in GTPases.

We examined the conservation of the G1–3 regions for each MglA group to explore the potential diversity of catalytic mechanisms utilized by MglA homologs. Structural and biochemical analyses of Group 1 MglA members in *T. thermophilus* and *M*yx*. xanthus* revealed an intrinsic arginine finger that is essential for GTPase activity (Miertzschke et al. 2011; Bulyha et al. 2013). This arginine residue is found adjacent to the conserved threonine of the G2 region (fig. 2*C*). We found that this arginine residue is conserved in all Group 1 MglA members and that the G1–3 motifs also show high conservation supporting a shared catalytic mechanism. Notably, the highly conserved G3 region in these proteins (TVPGQ) deviates from that of eukaryotic Ras superfamily members (DxxGQ) by having conserved threonine, valine, and proline residues in the first three positions. In contrast, members of Groups 2–5 lack this arginine in G2, and instead have a conserved threonine in its place (fig. 2*C*). Furthermore, Groups 2–5 share a conserved GTPGQ motif in the G3 region, which suggests that the MglA proteins in Groups 2–5 have a shared catalytic mechanism.

Accordingly, we examined the MglB sequences that are coupled to Groups 2–5 MglA members to determine whether they contain a conserved residue that could act as a transition state stabilizer similarly to the extrinsic arginine finger of eukaryotic GAPs, but we found very little sequence conservation other than a conserved aspartate glycine pair (fig. 2*C*). Unlike the glycine, the aspartate position is not highly conserved among MglB sequences coupled to Group 1 MglA members; however, there is a conserved aspartate two positions away (fig. 2*C*). Based on the MglA–MglB cocrystal structural analyses (Miertzschke et al. 2011), only the conserved aspartate of the Group 2–5 systems is predicted to be within interaction distance (5 Å) of MglA (supplementary fig. S1, Supplementary Material online); however, the function of the aspartate remains to be explored. In total, we conclude that the MglA members of Groups 2–5 likely share a similar catalytic mechanism that is distinct from Group 1 members and from systems characterized in eukaryotes.

### Classification of the Rup Family

**Fig. 3.—:**
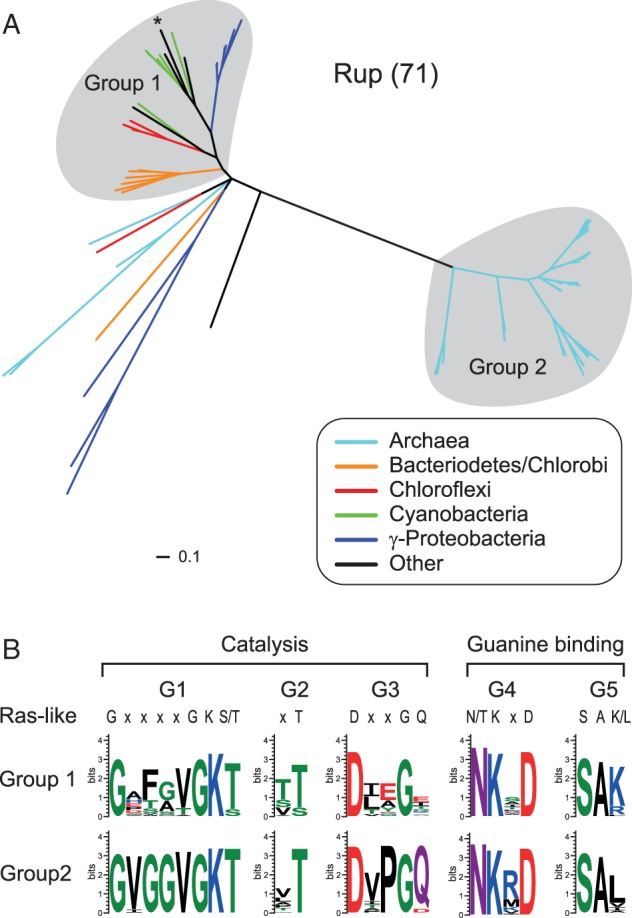
The Rup family contains two distinct groups. (*A*) A phylogenetic tree of small Rup GTPases. The Group 1 sequence from *Sorangium cellulosum* that is further described in figure 4A is marked with an asterisk. (*B*) Sequence logos of the G1–G5 motifs of Group 1 and Group 2 Rup sequences.

We have identified 71 small GTPases of the Rup subfamily, which are encoded in many the genomes of ancient and diverse taxa including Archaea, Bacteroidetes/Chlorobi, Chloroflexi, Cyanobacteria, Nitrospirae, α-Proteobacteria, γ-Proteobacteria, δ-Proteobacteria, and ε-Proteobacteria (fig. 3*A* and supplementary table S2, Supplementary Material online). We built a phylogenetic tree from a multiple sequence alignment of the 71 Rup family members. In this tree, we identified two distinct groups based on tree topology, taxonomy, and gene neighborhood analyses whereas the remaining Rup GTPases remain unclassified (fig. 3*A*). Group 1 Rup members show extensive taxonomic diversity and include species of Bacteriodetes, Chloroflexi, Cyanobacteria, and γ-Proteobacteria. Proteins of the same taxonomic lineage typically form monophyletic clades within the group, which supports that these systems are vertically inherited. Group 2 Rup members form a distinct clade that is exclusively composed of sequences encoded in a few species of Crenarchaeota. Interestingly, Group 2 Rup sequences are highly duplicated with as many as six copies per genome. No members of the Rup subfamily have been experimentally characterized, but one Group 1 Rup member in *Nostoc punctiforme* has been shown to be transcribed (Dong et al. 2007).

### Putative Rup Catalytic Mechanisms

Unlike MglA members, sequence analyses of Rup GTPases show that their G1–5 motifs have conservation patterns that are remarkably similar to the Ras superfamily GTPases in eukaryotes (fig. 3*B*). Surprisingly, Group 1 Rup members lack the conserved glutamine of the G3 motif that is generally essential for catalysis in Ras superfamily GTPases (Anand et al. 2013). However, this glutamine can be provided in trans by GAPs in eukaryotes (Anand et al. 2013) suggesting that this residue may similarly be provided in trans by an unknown GAP in the case of the Group 1 Rup proteins. Overall, the conservation analysis of the G1–5 motifs of Rup GTPases supports that they use catalytic mechanisms comparable to those that have been characterized in eukaryotic Ras superfamily members.

### Links to Signal Transduction Systems

**Fig. 4.—:**
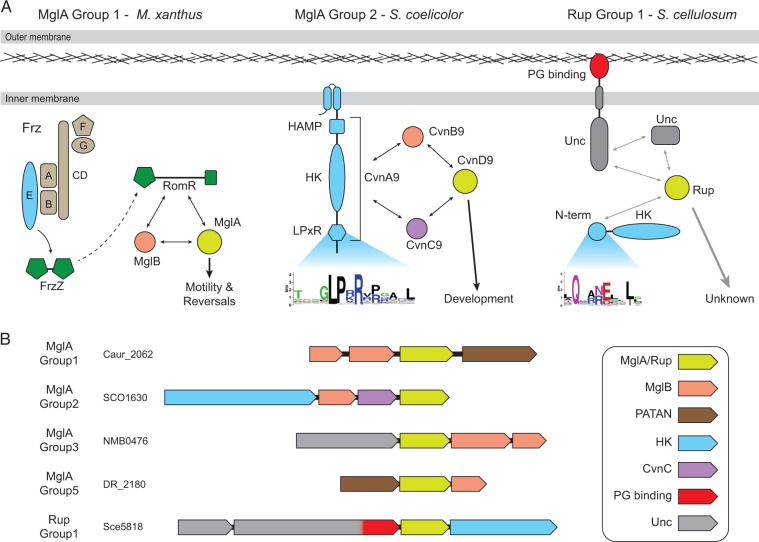
Links between small GTPases and other signal transduction systems. (*A*) Two characterized MglA interaction networks are shown in addition to a model of a Rup interaction network. The Group 1 MglA system of *Myx. xanthus* involves the Frz chemosensory system (FrzA–G and FrzZ), the RomR response regulator, and MglB. A dashed line indicates that no direct interaction has been shown between FrzZ and RomR. Cvn9 of *S. coelicolor* is a Group 2 MglA system. In addition to inner membrane spanning transmembrane helices, an HAMP domain, and a histidine kinase (HK) module composed of the autophosphorylated dimerization domain and the ATPase domain, CvnA homologs contain a conserved C-terminal LPxR motif shown by a sequence logo of the conservon histidine kinases. Although no Rup system interactions have been experimentally characterized, gray lines indicate hypothetical interactions based on homology to known interaction systems and component conservation. The sequence logo shows the conserved glutamine of the N-terminal domains (N-term) of the signal transduction components associated with the Group 1 Rup GTPases. Unc indicates proteins with conserved domains of unknown function that do not match current Pfam domain models. (*B*) Representative gene neighborhoods that support interaction between small GTPases and other prokaryotic signal transduction systems. The locus tag corresponding to the first gene of each neighborhood is provided.

Members of the Ras superfamily of small GTPases in prokaryotes have been linked to other signal transduction systems, in particular classic two component systems, through experimental and computational analyses. Specifically, MglA of *M*yx*. xanthus* has been shown to interact with three different response regulators that regulate cellular motility: AglZ, FrzS, and RomR (Yang et al. 2004; Mauriello et al. 2010; Keilberg et al. 2012; Zhang et al. 2012) (fig. 4*A*). Moreover, genetic evidence suggests that the Frz chemosensory system also links to MglA through RomR (Keilberg et al. 2012). The *B. bacteriovorus* MglA has been shown to function together with a RomR homolog to regulate predation (Milner et al. 2014). In these organisms, *mglA* is not located near genes encoding these interaction partners. Conversely, in the case of the actinobacterial conservons, *mglA* is encoded adjacent to the genes of its interaction partners.

Because the majority of the computationally identified GTPases remain functionally uncharacterized, we surveyed the genome context of the genes encoding MglA or Rup GTPases to identify potential interaction partners (supplementary table S2, Supplementary Material online). We found that Group 1, 4, and 5 MglA members vary widely in gene neighborhood composition; however, occasionally, some Group 1 and 5 members are encoded near genes encoding proteins containing a PATAN (PatA N-terminal domain) domain, which is named for its homology to the N-terminal domain of PatA (fig. 4*B* and supplementary table S2, Supplementary Material online), a response regulator involved in heterocyst formation in filamentous cyanobacteria (Liang et al. 1992). This is consistent with a previous genome context analysis that identified MglB homologs encoded close to PATAN-containing proteins (Makarova et al. 2006). We did not identify conserved proteins encoded near Group 4 *mglA* genes.

In contrast to Groups 1, 4, and 5, the gene neighborhoods of Group 2 and 3 MglA members are highly conserved (supplementary table S2, Supplementary Material online). Group 2 systems are part of the conservon of Actinobacteria, which is composed of four proteins: CvnA, a histidine kinase, CvnB, a homolog of MglB, CvnC, a protein of unknown function, and CvnD, a homolog of MglA (fig. 4*A*). Our phylogenetic analyses show that the histidine kinases of the conservon form a clade that is highly distinct from other Actinobacteria histidine kinases (supplementary fig. S2, Supplementary Material online). The conservon histidine kinases have a C-terminal extension that contains a conserved LPxR motif (fig. 4*A*). The conserved arginine of this motif is notable given the lack of a conserved arginine finger in Group 2 MglA or MglB sequences; however, we are cautious in speculating about the function of this residue based on our prediction that Groups 2–5 share a similar catalytic mechanism and this motif has not been found in proteins associated with Groups 3–5 MglA members. Given that the conservon does not include a response regulator protein, we posit that the Group 2 MglA protein is the output of this system, unlike classic two-component systems. Group 3 systems typically consist of a conserved protein of unknown function, an MglA homolog, and one or two MglB homologs (fig. 4*B* and supplementary table S2, Supplementary Material online). A multiple sequence alignment of the protein of unknown function reveals conserved N-terminal and C-terminal regions that do not match current Pfam domain models and are separated by a linker of variable length and composition. Fold prediction using the Phyre2 server (Kelley and Sternberg 2009) supports with reasonable certainty (confidence score of at least a 90%) that the N-terminal region is structurally similar to the receiver domain of response regulators and the C-terminal region contains a region structurally similar to a winged helix–turn–helix DNA-binding domain. This domain architecture suggests that Group 3 MglA proteins may be part of a signal transduction system regulating gene transcription.

We did not identify conserved proteins encoded in the gene neighborhoods of Group 2 Rup GTPases. However, Group 1 Rup GTPases are encoded in gene neighborhoods composed of three conserved genes in addition to the *rup* gene. These genes encode two uncharacterized proteins, one of which is predicted to form a coiled-coil cytoplasmic protein. The second protein contains a conserved domain of unknown function that is often followed by an inner membrane spanning α-helix and a C-terminal peptidoglycan-binding domain (Pfam: OmpA) supporting a role of this protein in membrane signaling (fig. 4 and supplementary table S2, Supplementary Material online). The fourth highly conserved gene in Group 1 Rup gene neighborhoods encodes a signal transduction output module, typically either a diguanylate cyclase/phosphodiesterase fusion protein involved in regulation of the accumulation of the nucleotide second messenger c-di-GMP or a histidine kinase (supplementary table S2, Supplementary Material online). These signal transduction proteins have an N-terminal region that includes an invariant glutamine residue in a multiple sequence alignment (fig. 4*A*). This glutamine is noteworthy given the absence of the catalytic glutamine in the G3 region of Group 1 Rup sequences, which we speculated to be provided in trans by an unknown GAP. Based on the predicted cellular localization of the four Group 1 Rup system components, we hypothesize that Group 1 Rup GTPases are part of a complex signal transduction system analogously to the actinobacterial conservon (fig. 4*B*).

### Evolutionary History of the Ras Superfamily of Small GTPases

**Fig. 5.—:**
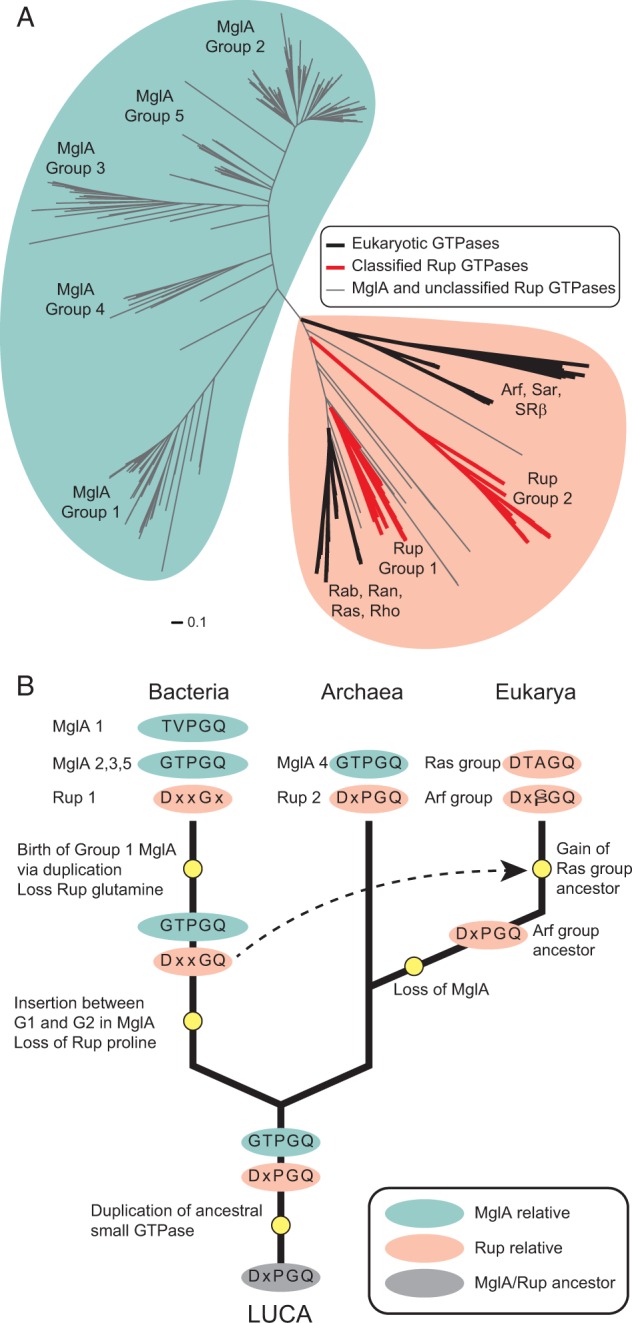
Origins of the Ras superfamily of small GTPases. (*A*) A phylogenetic tree built from a multiple sequence alignment of small prokaryotic and eukaryotic GTPases of the Ras superfamily. Locations of the seven subfamilies of eukaryotic Ras superfamily homologs are indicated in addition to the groups of MglA and Rup GTPases described in this study. (*B*) Evolutionary scenario of the observed distribution of Ras superfamily GTPases in extant organisms. Ras superfamily members are indicated by ovals containing the observed G3 motif of extant members and hypothesized G3 motifs of ancestral sequences. Important evolutionary events are indicated by yellow circles. Arf and Ras groups correspond to the Arf/Sar/SRβ, and Rab/Ran/Ras/Rho groups of eukaryotic small GTPases, respectively.

Previously computational analyses of small GTPases in prokaryotes only focused on a limited subset of small GTPases. The approach taken here is a more comprehensive analysis of 520 small GTPases in diverse species many of which were only recently sequenced. With this more comprehensive data set, we revisited the relationship between prokaryotic and eukaryotic GTPases and traced the evolutionary history that resulted in the emergence and distribution of the extant members of this highly versatile protein family. To this end, we used previously identified representatives of seven eukaryotic Ras GTPase subfamilies (Arf, Rab, Ran, Ras, Rho, Sar, and SRβ) (Dong et al. 2007) as queries in BLASTP analyses in order to identify additional eukaryotic homologs (Materials and Methods). These sequences were then included in a phylogenetic analysis of our previously identified prokaryotic small GTPases (fig. 5*A*).

In agreement with a previous analysis, we find that the eukaryotic proteins do not form a monophyletic clade (Dong et al. 2007; Yutin et al. 2009) (fig. 5*A*) and that the Rab/Ran/Ras/Rho proteins are very similar to the Group 1 Rup proteins, whereas the Arf/Sar/SRβ proteins are distant from MglA and Rup sequences. Of note, as seen in the analysis of prokaryotic small GTPases, the more comprehensive analysis including eukaryotic small GTPases still shows that there are only two major families of small GTPases: The MglA family, which is exclusive to prokaryotes, and the family containing all eukaryotic proteins as well as the Rup proteins.

This tree provides an overall framework for considering the evolution of small GTPases. First, MglA and Rup GTPases are found in diverse, ancient and deep branching taxonomic lineages, and the protein sequences from related organisms often group together within the corresponding phylogenetic trees, which is a reflection of vertical inheritance of these genes. Furthermore, the Group 4 MglA and Group 2 Rup proteins, which are found only in archaea, do not branch closely with their bacterial counterparts and vice versa, supporting the notion that these proteins were not acquired by a horizontal gene transfer event between bacteria and archaea. Based on these observations, we propose that both an MglA and a Rup ancestor were present in LUCA prior to the diversification of Bacteria and Archaea (fig. 5*B*). Subsequently, both ancestral proteins were vertically propagated in bacteria and archaea. Gene loss, which is a dominant evolutionary force shaping genomes (Wolf and Koonin 2013), is likely responsible for the absence of these GTPases in many extant prokaryotic lineages. The emergence of MglA prior to the diversification of LUCA is also consistent with the notion that MglB is an ancient protein and present in all three kingdoms of life (Koonin and Aravind 2000). Based on the sequence, structural, and phylogenetic analyses we hypothesize that the ancestors of MglA and Rup in LUCA were originally quite similar and that the diversification of these sequences was influenced by their interactions with various proteins including MglB in the case of MglA (fig. 5*B*). By parsimony, we posit that the ancestral GTPase in LUCA that was duplicated giving rise to the MglA and Rup families had a G3 region that was similar to the ancestors of both families, and most likely contained the conserved aspartate in G3 typical of nearly all P-loop GTPases (fig. 5*B*).

Several lines of evidence suggest that the eukaryotic small GTPases have two distinct origins. First, in the case of the Arf/Sar/SRβ group, the closest homologs are in archaea, that is, the Group 2 Rup proteins and Group 4 MglA proteins. Based on this phylogenetic analysis (fig. 5*A*) it seems likely that the ancestor of the Arf/Sar/SRβ group was vertically inherited from the archaeal ancestor of eukaryotes. There is emerging evidence that the archaeal ancestor of eukaryotes may have belonged to the ancient TACK (Thaumarchaeota, Aigarchaeota, Crenarchaeota, and Korarchaeota) superphylum (Martijn and Ettema 2013; Koonin and Yutin 2014). Intriguingly, Group 2 Rup sequences are found exclusively in the Crenarchaeota. With the exception of a single Group 4 MglA protein that is encoded in the genome of the only sequenced korarchaeotal genome, all Group 4 MglA proteins are exclusive to Euryarchaeota. Thus, the Arf/Sar/SRβ group could have originated from a Group 2 Rup or Group 4 MglA ancestor. Previously, it was indeed posited that the Arf/Sar/SRβ group originated from the MglA family (Dong et al. 2007). Although this possibility cannot be formally excluded, we strongly favor the hypothesis that the Arf/Sar/SRβ group and Group 2 Rup share a common ancestor given the similarities between the catalytic motifs of Group 2 Rup proteins and the Arf/Sar/SRβ group of GTPases. In particular, the conserved aspartate in the G3 region is highly conserved in all GTPases other than MglA. Furthermore, MglB homologs encoded in the genomes of eukaryotes have not been implicated in interactions with eukaryotic small GTPases (Koonin and Aravind 2000), which would be expected if the Arf/Sar/SRβ group shared a common ancestor with MglA. Thus, we propose that the *mglA* gene was lost in the ancestral eukaryote whereas the *mglB* gene remained and likely underwent neofunctionalization.

We previously noted that Rab/Ran/Ras/Rho are very similar to the Group 1 Rup sequences, which are found in distantly related bacteria (fig. 5*A*). This close grouping suggests that the Rab/Ran/Ras/Rho group has not evolved in a manner consistent with vertical inheritance. We hypothesize that this group originated due to a horizontal gene transfer event (fig. 5*B*), which is consistent with conclusions from previous analyses (Dong et al. 2007; Yutin et al. 2009). Interestingly, there is a Group 1 Rup member in *Mesorhizobium loti*, which supports that this system may have been present in other α-Proteobacteria, a member of which is the presumed ancestor of the mitochondrion (Esser et al. 2004). The subsequent expansion of the Arf/Sar/SRβ and Rab/Ran/Ras/Rho groups in eukaryotes suggests that these proteins and their diverse functions significantly contribute to the fitness of these organisms.

Group 2–5 MglA proteins are exclusively encoded in bacterial genomes with the exception of a few archaeal sequences predicted to have been acquired through horizontal gene transfer (supplementary table S2, Supplementary Material online). We find that Group 1 and Group 5 members, some of which are found in species from deep branching phyla including Aquificae, Chlorobi, and Deinococus/Thermus, are often encoded in the same genomes, and these sequences show evidence of vertical inheritance within each group (fig. 2*A*). These findings suggest that there was a duplication event of MglA within an ancestral bacterium. Bacterial MglA sequences contain an insertion between the G1 and G2 regions that is neither present in archaeal nor in eukaryotic small GTPases. Additionally, this insertion is absent in all Rup sequences. Therefore, the insertion event happened in an ancestral bacterium prior to the MglA duplication event. The similarity between the G3 region of bacterial Groups 2, 3, and 5 MglA sequences and the G3 region of archaeal Group 4 MglA sequences supports that they represent the most ancestral form of MglA and that the MglA duplication event in an ancestral bacterium gave rise to Group 1 MglA and members of this group then underwent rapid diversification as evidenced by their distinct catalytic mechanism.

## Discussion

Small GTPases of the Ras superfamily have been shown to play important roles in development and antibiotic resistance in addition to correctly localizing proteins in order to enable effective motility and predation. The versatility of these proteins in the regulation of diverse cellular processes mirrors their association with a variety of signal transduction systems. Our analysis identified an abundance of small GTPases in a wide range of prokaryotes, and the vast majority of these small GTPases remain uncharacterized experimentally. Importantly, these GTPases are also found in diverse pathogens, such as *Neisseria* spp and *M*yc*. smegmatis*. Furthermore, these GTPases are found in methanogenic archaea, which have a key role in the anthropogenic emission of the potent greenhouse gas methane (Shi et al. 2014). The study of these dynamic and versatile proteins in prokaryotes is an emerging field, and it is likely that future studies will continue to reveal their involvement in assorted functions.

By performing a comprehensive phylogenomic analysis of prokaryotic small GTPases we have been able to classify these proteins into discrete groups that are divided into two families, the MglA and Rup families. Furthermore, we have been able to use the classification data along with phylogenetic analyses to propose a simple scenario for the evolution of these proteins that begins with the presence of two small GTPases in LUCA that ultimately evolved into the small GTPases seen in extant organisms. In addition to providing an evolutionary framework for understanding the history of small GTPases, our analysis also identified conserved components predicted to interact with many of these as yet uncharacterized proteins. Thus, we have provided a rich source of information that lays the foundation for the continued experimental exploration of these exciting signal transduction systems.

## Supplementary Material

Supplementary tables S1–S4 and figures S1 and S2 are available at *Genome Biology and Evolution* online (http://www.gbe.oxfordjournals.org/).

Supplementary Data
