# Supplementary material for: Evolution and Diversity of the Ras Superfamily of Small GTPases in Prokaryotes
Source: Genome Biol Evol. 2014 Dec 4;7(1):57–70. doi: 10.1093/gbe/evu264 (PMC4316618; doi:10.1093/gbe/evu264)
Supplement: Supplementary Data [file supp_7_1_57__index.html]

Evolution and diversity of the Ras superfamily of small GTPases in prokaryotes — Evolution and Diversity of the Ras Superfamily of Small GTPases in Prokaryotes — Supplementary Data 

# Evolution and Diversity of the Ras Superfamily of Small GTPases in Prokaryotes

## Supplementary Data

files

**Files in this Data Supplement:**

- Supplementary Data - pdf file
- Supplementary Data - docx file
