# Supplementary material for: Evolution and Diversity of the Ras Superfamily of Small GTPases in Prokaryotes
Source: Genome Biol Evol. 2014 Dec 4;7(1):57–70. doi: 10.1093/gbe/evu264 (PMC4316618; doi:10.1093/gbe/evu264)
Supplement: Supplementary Data [file supp_evu264_WuichetSuppFINAL.pdf]

Supplementary Material

Evolution and diversity of the Ras superfamily of small GTPases in prokaryotes

Kristin Wuichet[1] & Lotte Søgaard-Andersen[1*]

[1]Department of Ecophysiology

Max Planck Institute for Terrestrial Microbiology

Karl-von-Frisch Str. 10

35043 Marburg, Germany

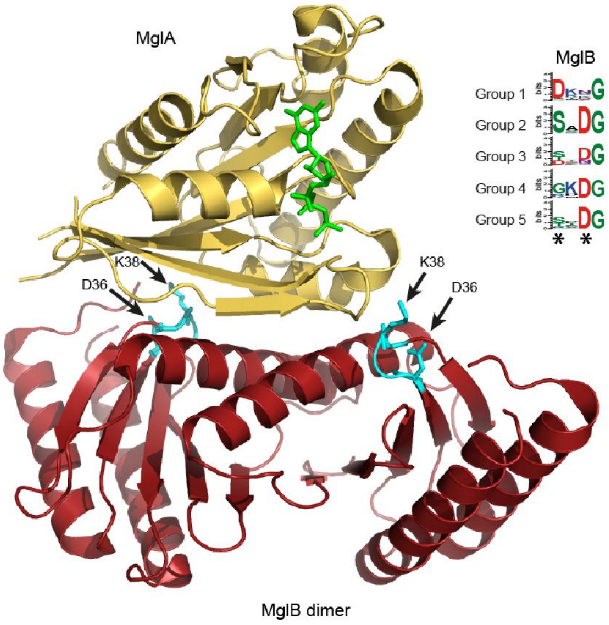

Fig. S1. Structure of MglA-MglB complex of *Thermus thermophilus*. GppNHp is indicated in green. The amino acid residues that correspond to the conserved Asp residues in MglB group 1 and groups 2-5 are indicated. Note that the latter residue is a Lys in *T. thermophilus* MglB, which is a group 1 member.

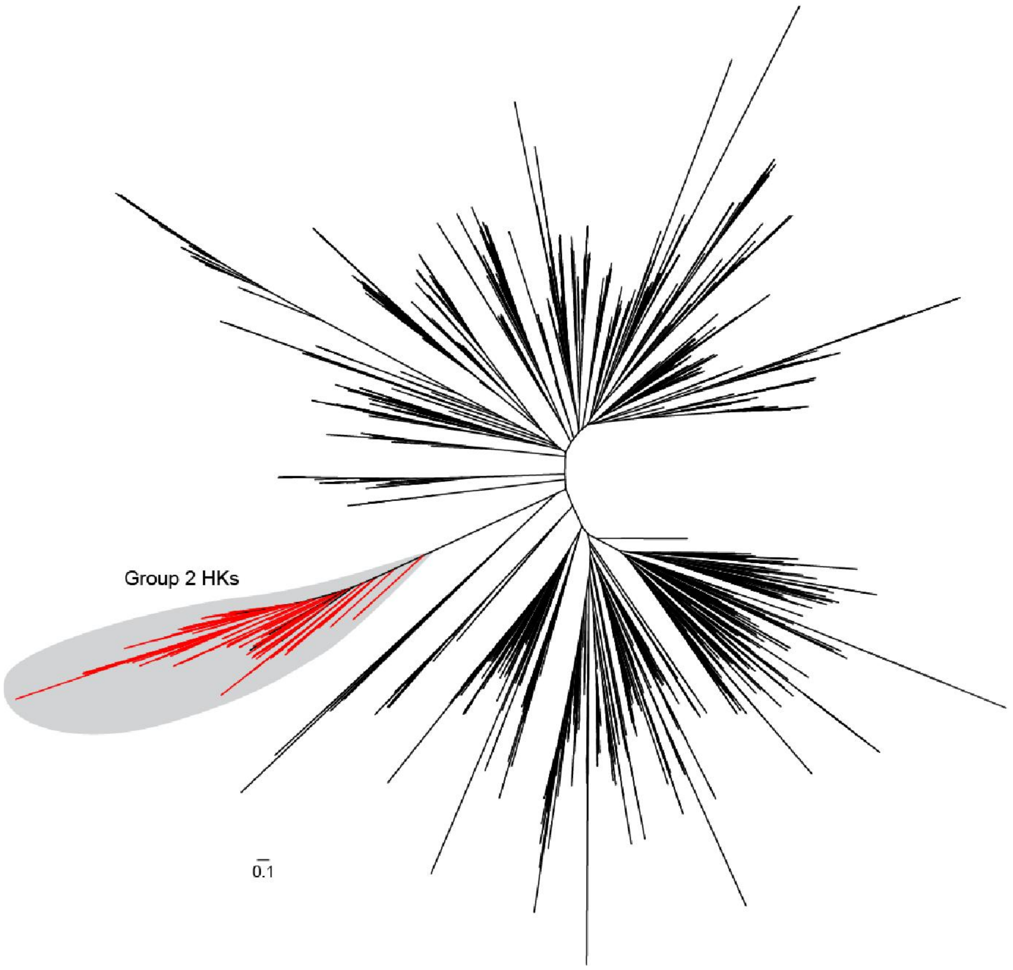

Fig. S2. Histidine kinases (HKs) encoded with the Group 2 conservon form a distinct subfamily. All sequences that contain a Pfam HATPase_c domain were collected from the genomes encoding Group 2 MglA sequences. The sequence regions corresponding to the HATPase_c domains were extracted and aligned using MAFFT with default settings. A tree was built from the alignment using FastTree with default settings. Branches in red indicate HKs encoded within four genes of a Group 2 *mglA* gene.

Table S1. Domain architecture analysis of 63 MglB (MXAN_1926) BLAST hits with an e-value of 0.0001 or better.

Pfam domain	HMM threshold[1]
Robl_LC7	58
None	5

[1] HMM threshold indicates the number of BLAST hits with sequences that match the corresponding Pfam domain model within the gathering threshold unless no domains are identified (None).

Table S2. Identified MglA and Rup GTPases as well as coupled MglB sequences. GTPase classification, taxonomy, genome, locus, gene neighborhood, and GTPase length are shown. The locus corresponds to the first sequence in the gene neighborhood. In the gene neighborhood analysis PATAN indicates a protein encoding a PATAN domain. DUF indicates a conserved protein of unknown function associated with Group 3 MglA sequences. DUFX and DUFY indicate two conserved proteins of unknown function associated with Group 1 Rup sequences. CvnCis a member of the actinobacterial conservon and matches the Pfam DUF742 domain. OmpA indicates a peptidoglycan binding domain. Gene fusions are indicated by a ":" symbol.

Table S2. Identified MglA and Rup GTPases as well as coupled MglB sequences. GTPase classification, taxonomy, genome, locus, gene neighborhood, and GTPase length are shown. The locus corresponds to the first sequence in the gene neighborhood. In the gene neighborhood analysis PATAN indicates a protein encoding a PATAN domain. DUF indicates a conserved protein of unknown function associated with Group 3 MglA sequences. DUFX and DUFY indicate two conserved proteins of unknown function associated with Group 1 Rup sequences. CvnCis a member of the actinobacterial conservon and matches the Pfam DUF742 domain. OmpA indicates a peptidoglycan binding domain. Gene fusions are indicated by a ":" symbol.

GTPase Group	Taxonomy	Genome	Locus	Gene Neighborhood	GTPase length
MglA Group 1	Acidobacteria	Candidatus Chloracidobacterium thermophilum B	Cabther_A0780	MglB>MglA>	196
MglA Group 1	Acidobacteria	Candidatus Koribacter versatilis Ellin345	Acid345_0708	MglA>	194
MglA Group 1	Aquificae	Aquifex aeolicus VF5	aq_1560	MglA>PATAN>	190
MglA Group 1	Aquificae	Desulfurobacterium thermolithotrophum DSM 11699	Dester_1068	<MglA<MglB	193
MglA Group 1	Aquificae	Hydrogenobacter thermophilus TK 6	HTH_0335	MglA>	186
MglA Group 1	Aquificae	Hydrogenobacter thermophilus TK 6	HTH_1830	<MglA<MglB	189
MglA Group 1	Aquificae	Hydrogenobacter thermophilus TK 6	HTH_1432	MglA>PATAN>	190
MglA Group 1	Aquificae	Sulfurihydrogenibium azorense Az Fu1	SULAZ_1271	MglB>MglA>	168
MglA Group 1	Aquificae	Thermocrinis albus DSM 14484	Thal_1140	<MglA<MglB	179
MglA Group 1	Aquificae	Thermocrinis albus DSM 14484	Thal_1033	MglA>	190
MglA Group 1	Aquificae	Thermovibrio ammonificans HB 1	Theam_1390	<MglA<MglB	193
MglA Group 1	Aquificae	Thermovibrio ammonificans HB 1	Theam_0113	<MglA--<MglB	202
MglA Group 1	Chlorobi	Chloroherpeton thalassium ATCC 35110	Ctha_0398	<PATAN:MglB-<MglA<MglB	196
MglA Group 1	Chlorobi	Chloroherpeton thalassium ATCC 35110	Ctha_2358	PATAN>MglB>MglA>	233
MglA Group 1	Chloroflexi	Chloroflexus aggregans DSM 9485	Cagg_2678	<PATAN<MglA<MglB<MglB	195
MglA Group 1	Chloroflexi	Chloroflexus aurantiacus J 10 fl	Caur_2059	<PATAN<MglA<MglB<MglB	195
MglA Group 1	Chloroflexi	Chloroflexus Y 400 fl	Chy400_2220	<PATAN<MglA<MglB<MglB	195
MglA Group 1	Chloroflexi	Herpetosiphon aurantiacus DSM 785	Haur_1007	MglA>	174
MglA Group 1	Chloroflexi	Herpetosiphon aurantiacus DSM 785	Haur_0930	<PATAN<MglA<MglB<MglB	191
MglA Group 1	Chloroflexi	Roseiflexus castenholzii DSM 13941	Rcas_4338	<PATAN<MglA<MglB	191
MglA Group 1	Chloroflexi	Roseiflexus RS 1	RoseRS_0199	MglB>MglA>PATAN>	191
MglA Group 1	Chloroflexi	Sphaerobacter thermophilus DSM 20745	Sthe_1565	MglB>-MglB>MglA>	191
MglA Group 1	Chloroflexi	Thermomicrobium roseum DSM 5159	trd_0862	MglB>-MglA>	191
MglA Group 1	Deferribacteres	Calditerrivibrio nitroreducens DSM 19672	Calni_1375	MglB>MglA>	192
MglA Group 1	Deferribacteres	Deferribacter desulfuricans SSM1	DEFDS_0024	<MglA<MglB	193
MglA Group 1	Deferribacteres	Flexistipes sinusarabici DSM 4947	Flexsi_0290	<MglA<MglB	193
MglA Group 1	Deinococcus-Thermus	Deinococcus deserti VCD115	Deide_17750	<MglA<MglB	196
MglA Group 1	Deinococcus-Thermus	Deinococcus geothermalis DSM 11300	Dgeo_1815	<MglA<MglB	196
MglA Group 1	Deinococcus-Thermus	Deinococcus maricopensis DSM 21211	Deima_2990	MglB>MglA>	196
MglA Group 1	Deinococcus-Thermus	Deinococcus proteolyticus MRP	Deipr_0324	MglB>MglA>	196
MglA Group 1	Deinococcus-Thermus	Deinococcus radiodurans R1	DR_0853	<MglA<MglB	196
MglA Group 1	Deinococcus-Thermus	Marinithermus hydrothermalis DSM 14884	Marky_0783	MglB>MglA>	196
MglA Group 1	Deinococcus-Thermus	Meiothermus ruber DSM 1279	Mrub_1713	<MglA<MglB	196
MglA Group 1	Deinococcus-Thermus	Meiothermus silvanus DSM 9946	Mesil_2006	<MglA<MglB	196
MglA Group 1	Deinococcus-Thermus	Oceanithermus profundus DSM 14977	Ocepr_1644	<MglA<MglB	196
MglA Group 1	Deinococcus-Thermus	Thermus scotoductus SA 01	TSC_c09700	<MglA<MglB	195
MglA Group 1	Deinococcus-Thermus	Thermus thermophilus HB27	TTC0766	MglB>MglA>	196
MglA Group 1	Deinococcus-Thermus	Thermus thermophilus HB8	TTHA1131	MglB>MglA>	196
MglA Group 1	Deinococcus-Thermus	Truepera radiovictrix DSM 17093	Trad_1082	<MglA	196
MglA Group 1	Dictyoglomi	Dictyoglomus thermophilum H 6 12	DICTH_1242	<MglA<MglB	195
MglA Group 1	Dictyoglomi	Dictyoglomus turgidum DSM 6724	Dtur_1356	<MglA<MglB	195
MglA Group 1	Fibrobacteres	Fibrobacter succinogenes S85	Fisuc_0226	<MglB<MglA	313
MglA Group 1	Gemmatimonadetes	Gemmatimonas aurantiaca T 27	GAU_1115	MglB>MglA>	225
MglA Group 1	Gemmatimonadetes	Gemmatimonas aurantiaca T 27	GAU_1409	PATAN>MglA>-PATAN>	229
MglA Group 1	Proteobacteria-beta	Dechloromonas aromatica RCB	Daro_0968	<MglA	214
MglA Group 1	Proteobacteria-delta	Anaeromyxobacter dehalogenans 2CP 1	A2cp1_3774	<MglA<MglB	195
MglA Group 1	Proteobacteria-delta	Anaeromyxobacter dehalogenans 2CP 1	A2cp1_4477	<MglA	230
MglA Group 1	Proteobacteria-delta	Anaeromyxobacter dehalogenans 2CP 1	A2cp1_1282	PATAN>-<MglA	355
MglA Group 1	Proteobacteria-delta	Anaeromyxobacter dehalogenans 2CP C	Adeh_3633	<MglA<MglB	195
MglA Group 1	Proteobacteria-delta	Anaeromyxobacter dehalogenans 2CP C	Adeh_4322	<MglA	230
MglA Group 1	Proteobacteria-delta	Anaeromyxobacter dehalogenans 2CP C	Adeh_1152	PATAN>-<MglA	356
MglA Group 1	Proteobacteria-delta	Anaeromyxobacter Fw109 5	Anae109_3758	<MglA<MglB	195
MglA Group 1	Proteobacteria-delta	Anaeromyxobacter Fw109 5	Anae109_4467	<MglA	271
MglA Group 1	Proteobacteria-delta	Anaeromyxobacter Fw109 5	Anae109_1199	PATAN>-<MglA	335
MglA Group 1	Proteobacteria-delta	Anaeromyxobacter K	AnaeK_3691	<MglA<MglB	195
MglA Group 1	Proteobacteria-delta	Anaeromyxobacter K	AnaeK_4458	<MglA	230
MglA Group 1	Proteobacteria-delta	Anaeromyxobacter K	AnaeK_1212	PATAN>-<MglA	352
MglA Group 1	Proteobacteria-delta	Bacteriovorax marinus SJ	BMS_0054	MglA>	196
MglA Group 1	Proteobacteria-delta	Bdellovibrio bacteriovorus HD100	Bd3734	MglA>	197
MglA Group 1	Proteobacteria-delta	Corallococcus coralloides DSM 2259	COCOR_01949	<MglA<MglB	195
MglA Group 1	Proteobacteria-delta	Corallococcus coralloides DSM 2259	COCOR_07291	<MglA	241
MglA Group 1	Proteobacteria-delta	Corallococcus coralloides DSM 2259	COCOR_05345	MglA><PATAN	378
MglA Group 1	Proteobacteria-delta	Desulfatibacillum alkenivorans AK 01	Dalk_2485	<MglA<MglB---<PATAN	201
MglA Group 1	Proteobacteria-delta	Desulfatibacillum alkenivorans AK 01	Dalk_0302	<MglA	216
MglA Group 1	Proteobacteria-delta	Desulfobacterium autotrophicum HRM2	HRM2_35580	<MglA<MglB	201
MglA Group 1	Proteobacteria-delta	Desulfobulbus propionicus DSM 2032	Despr_1662	MglB>MglA>	201
MglA Group 1	Proteobacteria-delta	Desulfococcus oleovorans Hxd3	Dole_2371	<PATAN<MglA	204
MglA Group 1	Proteobacteria-delta	Desulfotalea psychrophila LSv54	DP2776	<MglA<MglB	201
MglA Group 1	Proteobacteria-delta	Geobacter bemidjiensis Bem	Gbem_3962	<MglA<MglB	195
MglA Group 1	Proteobacteria-delta	Geobacter bemidjiensis Bem	Gbem_2315	<MglA	289
MglA Group 1	Proteobacteria-delta	Geobacter FRC 32	Geob_0373	<MglA<MglB	195
MglA Group 1	Proteobacteria-delta	Geobacter FRC 32	Geob_3508	<MglA	221
MglA Group 1	Proteobacteria-delta	Geobacter FRC 32	Geob_2389	MglA>	283
MglA Group 1	Proteobacteria-delta	Geobacter lovleyi SZ	Glov_3123	MglB>MglA>	195
MglA Group 1	Proteobacteria-delta	Geobacter lovleyi SZ	Glov_1322	MglA>	270
MglA Group 1	Proteobacteria-delta	Geobacter M18	GM18_4429	<MglA<MglB	195
MglA Group 1	Proteobacteria-delta	Geobacter M18	GM18_0303	<MglA	219
MglA Group 1	Proteobacteria-delta	Geobacter M18	GM18_1915	MglA>	288
MglA Group 1	Proteobacteria-delta	Geobacter M21	GM21_4048	<MglA<MglB	195
MglA Group 1	Proteobacteria-delta	Geobacter M21	GM21_2366	<MglA	223
MglA Group 1	Proteobacteria-delta	Geobacter M21	GM21_1908	MglA>	281
MglA Group 1	Proteobacteria-delta	Geobacter metallireducens GS 15	Gmet_3417	<MglA<MglB	195
MglA Group 1	Proteobacteria-delta	Geobacter metallireducens GS 15	Gmet_3475	<MglA	225
MglA Group 1	Proteobacteria-delta	Geobacter metallireducens GS 15	Gmet_1982	<MglA	289
MglA Group 1	Proteobacteria-delta	Geobacter sulfurreducens PCA	GSU0098	MglB>MglA>	195
MglA Group 1	Proteobacteria-delta	Geobacter sulfurreducens PCA	GSU1930	<MglA	291
MglA Group 1	Proteobacteria-delta	Geobacter uraniireducens Rf4	Gura_4331	MglB>MglA>	195
MglA Group 1	Proteobacteria-delta	Geobacter uraniireducens Rf4	Gura_2812	<MglA	305
MglA Group 1	Proteobacteria-delta	Haliangium ochraceum DSM 14365	Hoch_6866	<MglA<MglB	195
MglA Group 1	Proteobacteria-delta	Haliangium ochraceum DSM 14365	Hoch_2556	MglA>	220
MglA Group 1	Proteobacteria-delta	Myxococcus fulvus HW 1	LILAB_17450	<MglA<MglB	195
MglA Group 1	Proteobacteria-delta	Myxococcus fulvus HW 1	LILAB_12945	MglA>	239
MglA Group 1	Proteobacteria-delta	Myxococcus fulvus HW 1	LILAB_21165	PATAN><MglA	367
MglA Group 1	Proteobacteria-delta	Myxococcus xanthus DK 1622	MXAN_1925	<MglA<MglB	195
MglA Group 1	Proteobacteria-delta	Myxococcus xanthus DK 1622	MXAN_6703	<MglA	298
MglA Group 1	Proteobacteria-delta	Myxococcus xanthus DK 1622	MXAN_2693	PATAN><MglA	372

MglA Group 1	Proteobacteria-delta	Pelobacter carbinolicus DSM 2380	Pcar_0378	MglB>MglA>	196
MglA Group 1	Proteobacteria-delta	Pelobacter propionicus DSM 2379	Ppro_2940	<MglA<MglB	195
MglA Group 1	Proteobacteria-delta	Pelobacter propionicus DSM 2379	Ppro_0659	MglA>	293
MglA Group 1	Proteobacteria-delta	Sorangium cellulosum So ce 56	sce1492	<MglA	196
MglA Group 1	Proteobacteria-delta	Sorangium cellulosum So ce 56	sce7248	MglB>MglA>	196
MglA Group 1	Proteobacteria-delta	Sorangium cellulosum So ce 56	sce0386	<MglA	242
MglA Group 1	Proteobacteria-delta	Sorangium cellulosum So ce 56	sce2413	<MglA	433
MglA Group 1	Proteobacteria-delta	Stigmatella aurantiaca DW4 3 1	STAUR_2690	<MglA<MglB	195
MglA Group 1	Proteobacteria-delta	Stigmatella aurantiaca DW4 3 1	STAUR_1215	MglA>	237
MglA Group 1	Proteobacteria-delta	Stigmatella aurantiaca DW4 3 1	STAUR_3437	PATAN><MglA	379
MglA Group 1	Proteobacteria-delta	Syntrophobacter fumaroxidans MPOB	Sfum_0316	PATAN>MglB>MglA>	205
MglA Group 1	Proteobacteria-delta	Syntrophobacter fumaroxidans MPOB	Sfum_1651	PATAN>-MglB>MglA>	205
MglA Group 1	Proteobacteria-gamma	Hahella chejuensis KCTC 2396	HCH_00229	MglA>	216
MglA Group 1	Proteobacteria-gamma	Methylomicrobium alcaliphilum	MEALZ_0921	MglA>	239
MglA Group 1	Proteobacteria-gamma	Methylomonas methanica MC09	Metme_1144	<MglA	220
MglA Group 1	Thermodesulfobacteria	Thermodesulfatator indicus DSM 15286	Thein_0407	MglB>MglA>	214
MglA Group 2	Actinobacteria	Actinosynnema mirum DSM 43827	Amir_5416	<MglA<MglB<HK	195
MglA Group 2	Actinobacteria	Actinosynnema mirum DSM 43827	Amir_2066	HK>MglB>CvnC>MglA>	197
MglA Group 2	Actinobacteria	Actinosynnema mirum DSM 43827	Amir_0458	HK>MglB>CvnC>MglA>	202
MglA Group 2	Actinobacteria	Actinosynnema mirum DSM 43827	Amir_4813	<MglA<CvnC<MglB<HK	202
MglA Group 2	Actinobacteria	Amycolatopsis mediterranei U32	AMED_1430	HK>MglB>CvnC>MglA>	177
MglA Group 2	Actinobacteria	Amycolatopsis mediterranei U32	AMED_7466	<MglA<CvnC<MglB<HK	179
MglA Group 2	Actinobacteria	Amycolatopsis mediterranei U32	AMED_6401	HK>MglB>CvnC>MglA>	185
MglA Group 2	Actinobacteria	Amycolatopsis mediterranei U32	AMED_2491	HK>MglB>CvnC>MglA>	187
MglA Group 2	Actinobacteria	Amycolatopsis mediterranei U32	AMED_8921	<MglA<CvnC<MglB<HK	199
MglA Group 2	Actinobacteria	Amycolatopsis mediterranei U32	AMED_7677	<MglA<CvnC<MglB<HK	200
MglA Group 2	Actinobacteria	Amycolatopsis mediterranei U32	AMED_8527	<CvnC<MglB<MglA<CvnC<MglB<HK	202
MglA Group 2	Actinobacteria	Amycolatopsis mediterranei U32	AMED_5268	<MglA<CvnC<MglB<HK	208
MglA Group 2	Actinobacteria	Catenulispora acidiphila DSM 44928	Caci_1060	HK>MglB>CvnC>MglA>	186
MglA Group 2	Actinobacteria	Catenulispora acidiphila DSM 44928	Caci_2006	<MglA	186
MglA Group 2	Actinobacteria	Catenulispora acidiphila DSM 44928	Caci_2445	HK>MglB>CvnC>MglA>	194
MglA Group 2	Actinobacteria	Catenulispora acidiphila DSM 44928	Caci_3314	HK>MglB>MglA>	195
MglA Group 2	Actinobacteria	Catenulispora acidiphila DSM 44928	Caci_5183	HK>MglB>MglA>	195
MglA Group 2	Actinobacteria	Catenulispora acidiphila DSM 44928	Caci_4180	HK>MglB>CvnC>MglA>	197
MglA Group 2	Actinobacteria	Catenulispora acidiphila DSM 44928	Caci_7862	HK>MglB>CvnC>MglA>	202
MglA Group 2	Actinobacteria	Catenulispora acidiphila DSM 44928	Caci_0363	HK>MglB>CvnC>MglA>	211
MglA Group 2	Actinobacteria	Catenulispora acidiphila DSM 44928	Caci_2531	<MglA<CvnC<MglB<HK	213
MglA Group 2	Actinobacteria	Cellulomonas fimi ATCC 484	Celf_3591	<MglA<CvnC<MglB<HK	203
MglA Group 2	Actinobacteria	Cellulomonas flavigena DSM 20109	Cfla_3172	<MglA<CvnC<MglB<HK	203
MglA Group 2	Actinobacteria	Cellvibrio gilvus ATCC 13127	Celgi_0188	HK>MglB>CvnC>MglA>	203
MglA Group 2	Actinobacteria	Frankia alni ACN14a	FRAAL0484	<MglA<CvnC<MglB<HK	178
MglA Group 2	Actinobacteria	Frankia alni ACN14a	FRAAL1811	<MglA<CvnC<MglB<HK	179
MglA Group 2	Actinobacteria	Frankia alni ACN14a	FRAAL1145	<MglA<CvnC<MglB<HK	194
MglA Group 2	Actinobacteria	Frankia alni ACN14a	FRAAL4394	<MglA<CvnC<MglB<HK	236
MglA Group 2	Actinobacteria	Frankia CcI3	Francci3_0641	<MglA<CvnC<MglB<HK	198
MglA Group 2	Actinobacteria	Frankia CcI3	Francci3_2857	<MglA<CvnC<MglB<HK	206
MglA Group 2	Actinobacteria	Frankia CcI3	Francci3_4330	HK>MglB>CvnC>MglA>	214
MglA Group 2	Actinobacteria	Frankia EAN1pec	Franean1_0389	HK>MglB>CvnC>MglA>	197
MglA Group 2	Actinobacteria	Frankia EAN1pec	Franean1_5990	HK>MglB>CvnC>MglA>	198
MglA Group 2	Actinobacteria	Frankia EAN1pec	Franean1_5470	HK>MglB>CvnC>MglA>	204
MglA Group 2	Actinobacteria	Frankia EAN1pec	Franean1_5487	HK>MglB>MglA>	206
MglA Group 2	Actinobacteria	Frankia EAN1pec	Franean1_2384	HK>MglB>CvnC>MglA>	207
MglA Group 2	Actinobacteria	Frankia EuI1c	FraEuI1c_6174	<MglA<CvnC<MglB<HK	198
MglA Group 2	Actinobacteria	Frankia EuI1c	FraEuI1c_4569	<MglA<CvnC<MglB<HK	206
MglA Group 2	Actinobacteria	Frankia EuI1c	FraEuI1c_4759	<MglA<MglB<HK	208
MglA Group 2	Actinobacteria	Frankia EuI1c	FraEuI1c_1914	<MglA<CvnC<MglB<HK	212
MglA Group 2	Actinobacteria	Frankia EuI1c	FraEuI1c_1358	HK>MglB>CvnC>MglA>	213
MglA Group 2	Actinobacteria	Frankia EuI1c	FraEuI1c_2066	HK>MglB>CvnC>MglA>	228
MglA Group 2	Actinobacteria	Frankia symbiont of Datisca glomerata	FsymDg_3901	HK>MglB>CvnC>MglA>	198
MglA Group 2	Actinobacteria	Frankia symbiont of Datisca glomerata	FsymDg_0499	<MglA<CvnC<MglB<HK	214
MglA Group 2	Actinobacteria	Isoptericola variabilis 225	Isova_2835	<MglA<CvnC<MglB<HK	317
MglA Group 2	Actinobacteria	Jonesia denitrificans DSM 20603	Jden_0272	HK>MglB>CvnC>MglA>	205
MglA Group 2	Actinobacteria	Kineococcus radiotolerans SRS30216	Krad_3848	HK>MglB>CvnC>MglA>	203
MglA Group 2	Actinobacteria	Kineococcus radiotolerans SRS30216	Krad_0313	<MglA<CvnC<MglB<HK	207
MglA Group 2	Actinobacteria	Kitasatospora setae KM 6054	KSE_42780	<MglA<CvnC<MglB<HK	189
MglA Group 2	Actinobacteria	Kitasatospora setae KM 6054	KSE_62390	HK>MglB>CvnC>MglA>	197
MglA Group 2	Actinobacteria	Kitasatospora setae KM 6054	KSE_62800	HK>MglB>CvnC>MglA>	202
MglA Group 2	Actinobacteria	Kitasatospora setae KM 6054	KSE_37820	HK>MglB>CvnC>MglA>	203
MglA Group 2	Actinobacteria	Kitasatospora setae KM 6054	KSE_50420	HK>MglB>CvnC>MglA>	213
MglA Group 2	Actinobacteria	Kitasatospora setae KM 6054	KSE_13240	<MglA<CvnC<MglB<HK	239
MglA Group 2	Actinobacteria	Kitasatospora setae KM 6054	KSE_51280	<MglA<CvnC<MglB<HK<MglA<CvnC<MglB<HK	195, 195
MglA Group 2	Actinobacteria	Micromonospora aurantiaca ATCC 27029	Micau_0788	HK>MglB>CvnC>MglA>	197
MglA Group 2	Actinobacteria	Micromonospora aurantiaca ATCC 27029	Micau_5057	<MglA<CvnC<MglB<HK	200
MglA Group 2	Actinobacteria	Micromonospora aurantiaca ATCC 27029	Micau_4285	<MglA<CvnC<MglB<HK	202
MglA Group 2	Actinobacteria	Micromonospora aurantiaca ATCC 27029	Micau_5471	<MglA<CvnC<MglB<HK	205
MglA Group 2	Actinobacteria	Micromonospora aurantiaca ATCC 27029	Micau_4102	<MglA<MglB<HK	209
MglA Group 2	Actinobacteria	Micromonospora aurantiaca ATCC 27029	Micau_5762	HK>MglB>CvnC>MglA>	253
MglA Group 2	Actinobacteria	Micromonospora L5	ML5_1031	HK>MglB>CvnC>MglA>	197
MglA Group 2	Actinobacteria	Micromonospora L5	ML5_3251	HK>MglB>CvnC>MglA>	200
MglA Group 2	Actinobacteria	Micromonospora L5	ML5_4015	HK>MglB>CvnC>MglA>	202
MglA Group 2	Actinobacteria	Micromonospora L5	ML5_0564	<MglA<CvnC<MglB<HK	205
MglA Group 2	Actinobacteria	Micromonospora L5	ML5_2730	<MglA<CvnC<MglB<HK	253
MglA Group 2	Actinobacteria	Mycobacterium africanum GM041182	MAF_33770	<MglA<CvnC<MglB<HK	193
MglA Group 2	Actinobacteria	Mycobacterium avium 104	MAV_4328	<MglA<CvnC<MglB<HK	195
MglA Group 2	Actinobacteria	Mycobacterium avium paratuberculosis K 10	MAP3469c	<MglA<CvnC<MglB<HK	195
MglA Group 2	Actinobacteria	Mycobacterium bovis AF2122 97	Mb3397c	<MglA<CvnC<MglB<HK	193
MglA Group 2	Actinobacteria	Mycobacterium bovis BCG Mexico	BCGMEX_3432c	<MglA<CvnC<MglB<HK	193
MglA Group 2	Actinobacteria	Mycobacterium bovis BCG Pasteur 1173P2	BCG_3434c	<MglA<CvnC<MglB<HK	193
MglA Group 2	Actinobacteria	Mycobacterium bovis BCG Tokyo 172	JTY_3434	<MglA<CvnC<MglB<HK	193
MglA Group 2	Actinobacteria	Mycobacterium canettii CIPT 140010059	MCAN_33881	<MglA<CvnC<MglB<HK	193
MglA Group 2	Actinobacteria	Mycobacterium gilvum PYR GCK	Mflv_4886	<MglA<CvnC<MglB<HK	192
MglA Group 2	Actinobacteria	Mycobacterium intracellulare MOTT 02	OCO_41930	<MglA<CvnC<MglB<HK	207
MglA Group 2	Actinobacteria	Mycobacterium intracellulare MOTT 64	OCQ_43200	<MglA<CvnC<MglB<HK	188
MglA Group 2	Actinobacteria	Mycobacterium JDM601	JDM601_3197	<MglA<CvnC<MglB<HK	197
MglA Group 2	Actinobacteria	Mycobacterium JLS	Mjls_1216	HK>MglB>CvnC>MglA>	193
MglA Group 2	Actinobacteria	Mycobacterium KMS	Mkms_1206	HK>MglB>CvnC>MglA>	193
MglA Group 2	Actinobacteria	Mycobacterium marinum M	MMAR_1165	HK>MglB>CvnC>MglA>	200
MglA Group 2	Actinobacteria	Mycobacterium MCS	Mmcs_1189	HK>MglB>CvnC>MglA>	193
MglA Group 2	Actinobacteria	Mycobacterium rhodesiae NBB3	MycrhN_0533	<MglA<CvnC<MglB<HK	193
MglA Group 2	Actinobacteria	Mycobacterium smegmatis MC2 155	MSMEG_1637	HK>MglB>CvnC>MglA>	193
MglA Group 2	Actinobacteria	Mycobacterium Spyr1	Mspyr1_43020	<MglA<CvnC<MglB<HK	192

MglA Group 2	Actinobacteria	Mycobacterium tuberculosis CDC1551	MT3470	<MglA<CvnC<MglB<HK	193
MglA Group 2	Actinobacteria	Mycobacterium tuberculosis F11	TBFG_13398	<MglA<CvnC<MglB<HK	193
MglA Group 2	Actinobacteria	Mycobacterium tuberculosis H37Ra	MRA_3402	<MglA<CvnC<MglB<HK	193
MglA Group 2	Actinobacteria	Mycobacterium tuberculosis H37Rv	Rv3362c	<MglA<CvnC<MglB<HK	193
MglA Group 2	Actinobacteria	Mycobacterium tuberculosis KZN 1435	TBMG_03413	<MglA<CvnC<MglB<HK	193
MglA Group 2	Actinobacteria	Mycobacterium tuberculosis KZN 4207	TBSG_03436	<MglA<CvnC<MglB<HK	193
MglA Group 2	Actinobacteria	Mycobacterium ulcerans Agy99	MUL_2847	<MglA<CvnC<MglB<HK	201
MglA Group 2	Actinobacteria	Mycobacterium vanbaalenii PYR 1	Mvan_1536	HK>MglB>CvnC>MglA>	188
MglA Group 2	Actinobacteria	Nocardia cyriacigeorgica GUH 2	NOCYR_0682	HK>MglB>CvnC>MglA>	176
MglA Group 2	Actinobacteria	Nocardia cyriacigeorgica GUH 2	NOCYR_3415	<MglA<CvnC<MglB<HK	187
MglA Group 2	Actinobacteria	Nocardia cyriacigeorgica GUH 2	NOCYR_3222	HK>MglB>CvnC>MglA>	189
MglA Group 2	Actinobacteria	Nocardia cyriacigeorgica GUH 2	NOCYR_4743	HK>MglB>CvnC>MglA>	208
MglA Group 2	Actinobacteria	Nocardia farcinica IFM 10152	nfa6670	HK>MglB>CvnC>MglA>	176
MglA Group 2	Actinobacteria	Nocardia farcinica IFM 10152	nfa23510	HK>MglB>CvnC>MglA>	183
MglA Group 2	Actinobacteria	Nocardia farcinica IFM 10152	nfa49650	HK>MglB>CvnC>MglA>	194
MglA Group 2	Actinobacteria	Nocardia farcinica IFM 10152	nfa53060	HK>MglB>CvnC>MglA>	201
MglA Group 2	Actinobacteria	Nocardia farcinica IFM 10152	nfa26780	<MglA<CvnC<MglB<HK	204
MglA Group 2	Actinobacteria	Nocardiopsis dassonvillei DSM 43111	Ndas_2200	HK>MglB>CvnC>MglA>	191
MglA Group 2	Actinobacteria	Nocardiopsis dassonvillei DSM 43111	Ndas_0275	<MglA<CvnC<MglB<HK	193
MglA Group 2	Actinobacteria	Nocardiopsis dassonvillei DSM 43111	Ndas_0813	HK>MglB>CvnC>MglA>	197
MglA Group 2	Actinobacteria	Nocardiopsis dassonvillei DSM 43111	Ndas_3047	<CvnC<HK--<MglA<CvnC<MglB<HK	198
MglA Group 2	Actinobacteria	Nocardiopsis dassonvillei DSM 43111	Ndas_0489	HK>MglB>CvnC>MglA>	201
MglA Group 2	Actinobacteria	Nocardiopsis dassonvillei DSM 43111	Ndas_3607	<MglA	204
MglA Group 2	Actinobacteria	Nocardiopsis dassonvillei DSM 43111	Ndas_2752	<MglA<CvnC<MglB<HK	218
MglA Group 2	Actinobacteria	Nocardiopsis dassonvillei DSM 43111	Ndas_1138	HK>MglB>CvnC>MglA>	236
MglA Group 2	Actinobacteria	Pseudonocardia dioxanivorans CB1190	Psed_5724	HK>MglB>CvnC>MglA>	198
MglA Group 2	Actinobacteria	Rhodococcus erythropolis PR4	RER_45440	HK>MglB>CvnC>MglA>	184
MglA Group 2	Actinobacteria	Rhodococcus opacus B4	ROP_34920	HK>MglB>CvnC>MglA>	183
MglA Group 2	Actinobacteria	Rhodococcus opacus B4	ROP_54240	<MglA<MglB<HK	206
MglA Group 2	Actinobacteria	Saccharomonospora viridis DSM 43017	Svir_37470	HK>MglB>CvnC>MglA>	190
MglA Group 2	Actinobacteria	Saccharomonospora viridis DSM 43017	Svir_06940	HK>MglB>CvnC>MglA>	191
MglA Group 2	Actinobacteria	Saccharomonospora viridis DSM 43017	Svir_34240	<CvnC<MglB<MglA<CvnC<MglB<HK	195
MglA Group 2	Actinobacteria	Saccharomonospora viridis DSM 43017	Svir_02250	HK>MglB>CvnC>MglA>	197
MglA Group 2	Actinobacteria	Saccharomonospora viridis DSM 43017	Svir_33540	<MglA<CvnC<MglB<HK	199
MglA Group 2	Actinobacteria	Saccharomonospora viridis DSM 43017	Svir_24580	<MglA<CvnC<MglB<HK	204
MglA Group 2	Actinobacteria	Saccharopolyspora erythraea NRRL 2338	SACE_6336	HK>MglB>CvnC>MglA>	200
MglA Group 2	Actinobacteria	Saccharopolyspora erythraea NRRL 2338	SACE_0557	HK>MglB>CvnC>MglA>	202
MglA Group 2	Actinobacteria	Saccharopolyspora erythraea NRRL 2338	SACE_1953	HK>MglB>CvnC>MglA>	208
MglA Group 2	Actinobacteria	Saccharopolyspora erythraea NRRL 2338	SACE_1149	HK>MglB>CvnC>MglA>	210
MglA Group 2	Actinobacteria	Saccharopolyspora erythraea NRRL 2338	SACE_0690	HK>MglB>CvnC>MglA>	223
MglA Group 2	Actinobacteria	Salinispora arenicola CNS 205	Sare_0760	HK>MglB>CvnC>MglA>	196
MglA Group 2	Actinobacteria	Salinispora arenicola CNS 205	Sare_3977	<MglA<CvnC<MglB<HK	200
MglA Group 2	Actinobacteria	Salinispora arenicola CNS 205	Sare_3225	<MglA<CvnC<MglB<HK	201
MglA Group 2	Actinobacteria	Salinispora arenicola CNS 205	Sare_1289	HK>MglB>CvnC>MglA>	203
MglA Group 2	Actinobacteria	Salinispora arenicola CNS 205	Sare_4263	<MglA<CvnC<MglB<HK	205
MglA Group 2	Actinobacteria	Salinispora tropica CNB 440	Strop_3596	<MglA<CvnC<MglB<HK	199
MglA Group 2	Actinobacteria	Salinispora tropica CNB 440	Strop_0816	HK>MglB>CvnC>MglA>	203
MglA Group 2	Actinobacteria	Salinispora tropica CNB 440	Strop_3872	<MglA<CvnC<MglB<HK	205
MglA Group 2	Actinobacteria	Sanguibacter keddieii DSM 10542	Sked_01800	HK>MglB>CvnC>MglA>	189
MglA Group 2	Actinobacteria	Stackebrandtia nassauensis DSM 44728	Snas_5269	<MglA<CvnC<MglB<HK	197
MglA Group 2	Actinobacteria	Stackebrandtia nassauensis DSM 44728	Snas_1145	HK>MglB>CvnC>MglA>	199
MglA Group 2	Actinobacteria	Streptomyces avermitilis MA 4680	SAV_3877	HK>MglB>CvnC>MglA>	180
MglA Group 2	Actinobacteria	Streptomyces avermitilis MA 4680	SAV_1630	<MglA<CvnC<MglB<HK	182
MglA Group 2	Actinobacteria	Streptomyces avermitilis MA 4680	SAV_2179	HK>MglB>CvnC>MglA>	191
MglA Group 2	Actinobacteria	Streptomyces avermitilis MA 4680	SAV_2188	HK>MglB>CvnC>MglA>	194
MglA Group 2	Actinobacteria	Streptomyces avermitilis MA 4680	SAV_1252	HK>MglB>CvnC>MglA>	199
MglA Group 2	Actinobacteria	Streptomyces avermitilis MA 4680	SAV_1577	<MglB-<MglA<CvnC<MglB<HK	201
MglA Group 2	Actinobacteria	Streptomyces avermitilis MA 4680	SAV_6958	HK>MglB>CvnC>-MglA>	201
MglA Group 2	Actinobacteria	Streptomyces avermitilis MA 4680	SAV_6702	HK>MglB>CvnC>MglA>	206
MglA Group 2	Actinobacteria	Streptomyces avermitilis MA 4680	SAV_2959	<MglA<CvnC<MglB<HK	208
MglA Group 2	Actinobacteria	Streptomyces avermitilis MA 4680	SAV_2694	HK>MglB>CvnC>MglA>HK>MglB>CvnC>MglA>	192, 174
MglA Group 2	Actinobacteria	Streptomyces bingchenggensis BCW 1	SBI_04566	HK>MglB>CvnC>MglA>	196
MglA Group 2	Actinobacteria	Streptomyces bingchenggensis BCW 1	SBI_01995	<MglA<CvnC<MglB<HK	197
MglA Group 2	Actinobacteria	Streptomyces bingchenggensis BCW 1	SBI_01961	HK>MglB>CvnC>MglA>	198
MglA Group 2	Actinobacteria	Streptomyces bingchenggensis BCW 1	SBI_03250	<MglA<CvnC<MglB<HK	203
MglA Group 2	Actinobacteria	Streptomyces bingchenggensis BCW 1	SBI_03866	<MglA<CvnC<MglB<HK	208
MglA Group 2	Actinobacteria	Streptomyces bingchenggensis BCW 1	SBI_04299	<MglA<CvnC<MglB<HK	209
MglA Group 2	Actinobacteria	Streptomyces bingchenggensis BCW 1	SBI_08883	HK>MglB>CvnC>MglA>	213
MglA Group 2	Actinobacteria	Streptomyces bingchenggensis BCW 1	SBI_05493	HK>MglB>CvnC>MglA>	214
MglA Group 2	Actinobacteria	Streptomyces bingchenggensis BCW 1	SBI_07326	<MglA<CvnC<MglB<HK	352
MglA Group 2	Actinobacteria	Streptomyces bingchenggensis BCW 1	SBI_03518	HK>MglB>CvnC>MglA>HK>MglB>CvnC>MglA>	173, 173
MglA Group 2	Actinobacteria	Streptomyces cattleya NRRL 8057	SCAT_p0473	HK>MglB>CvnC>MglA>	196
MglA Group 2	Actinobacteria	Streptomyces cattleya NRRL 8057	SCAT_0732	<MglA<CvnC<MglB<HK	202
MglA Group 2	Actinobacteria	Streptomyces cattleya NRRL 8057	SCAT_4134	HK>MglB>CvnC>MglA>	209
MglA Group 2	Actinobacteria	Streptomyces cattleya NRRL 8057	SCAT_3209	HK>MglB>CvnC>MglA>	217
MglA Group 2	Actinobacteria	Streptomyces cattleya NRRL 8057	SCAT_4368	<MglA<CvnC<MglB<HK<MglA<CvnC<MglB<HK	192, 192
MglA Group 2	Actinobacteria	Streptomyces coelicolor A3 2	SCO1627	<MglA<CvnC<MglB<HK	176
MglA Group 2	Actinobacteria	Streptomyces coelicolor A3 2	SCO7463	HK>MglB>CvnC>MglA>	176
MglA Group 2	Actinobacteria	Streptomyces coelicolor A3 2	SCO0585	<MglA<CvnC<MglB<HK	178
MglA Group 2	Actinobacteria	Streptomyces coelicolor A3 2	SCO6066	<MglA<CvnC<MglB<HK	181
MglA Group 2	Actinobacteria	Streptomyces coelicolor A3 2	SCO2879	HK>MglB>CvnC>MglA>	182
MglA Group 2	Actinobacteria	Streptomyces coelicolor A3 2	SCO7419	<MglA<CvnC<MglB<HK	183
MglA Group 2	Actinobacteria	Streptomyces coelicolor A3 2	SCO6794	HK>MglB>CvnC>MglA>	184
MglA Group 2	Actinobacteria	Streptomyces coelicolor A3 2	SCO5289	HK>MglB>CvnC>MglA>	191
MglA Group 2	Actinobacteria	Streptomyces coelicolor A3 2	SCO6940	<MglA<CvnC<MglB<HK	192
MglA Group 2	Actinobacteria	Streptomyces coelicolor A3 2	SCO1399	<MglA<CvnC<MglB<HK	201
MglA Group 2	Actinobacteria	Streptomyces coelicolor A3 2	SCO1155	<MglB-<MglA<CvnC<MglB<HK	204
MglA Group 2	Actinobacteria	Streptomyces coelicolor A3 2	SCO5537	<MglA<CvnC<MglB<HK<MglA<CvnC<MglB<HK	174, 174
MglA Group 2	Actinobacteria	Streptomyces flavogriseus ATCC 33331	Sfla_6226	<MglA<CvnC<MglB<HK	196
MglA Group 2	Actinobacteria	Streptomyces flavogriseus ATCC 33331	Sfla_4530	HK>MglB>CvnC>MglA>	201
MglA Group 2	Actinobacteria	Streptomyces flavogriseus ATCC 33331	Sfla_5467	HK>MglB>CvnC>MglA>	201
MglA Group 2	Actinobacteria	Streptomyces flavogriseus ATCC 33331	Sfla_0609	<MglA<CvnC<MglB<HK	203
MglA Group 2	Actinobacteria	Streptomyces flavogriseus ATCC 33331	Sfla_0731	<MglA<CvnC<MglB<HK	204
MglA Group 2	Actinobacteria	Streptomyces flavogriseus ATCC 33331	Sfla_1393	<MglA<CvnC<MglB<HK	204
MglA Group 2	Actinobacteria	Streptomyces flavogriseus ATCC 33331	Sfla_0232	<MglB<MglA<CvnC<MglB<HK	211
MglA Group 2	Actinobacteria	Streptomyces flavogriseus ATCC 33331	Sfla_2001	<MglA<CvnC<MglB<HK	216
MglA Group 2	Actinobacteria	Streptomyces flavogriseus ATCC 33331	Sfla_2696	HK>MglB>CvnC>MglA>	219
MglA Group 2	Actinobacteria	Streptomyces flavogriseus ATCC 33331	Sfla_1755	HK>MglB>CvnC>MglA>HK>MglB>CvnC>MglA>	193, 193
MglA Group 2	Actinobacteria	Streptomyces griseus NBRC 13350	SGR_3490	HK>MglB>CvnC>MglA>	196
MglA Group 2	Actinobacteria	Streptomyces griseus NBRC 13350	SGR_6126	HK>MglB>CvnC>MglA>	202
MglA Group 2	Actinobacteria	Streptomyces griseus NBRC 13350	SGR_1060	<MglA<CvnC<MglB<HK	204

MglA Group 2	Actinobacteria	Streptomyces griseus NBRC 13350	SGR_4393	<MglA<CvnC<MglB<HK	204
MglA Group 2	Actinobacteria	Streptomyces griseus NBRC 13350	SGR_932	<MglA<CvnC<MglB<HK	207
MglA Group 2	Actinobacteria	Streptomyces griseus NBRC 13350	SGR_3103	HK>MglB>CvnC>MglA>	214
MglA Group 2	Actinobacteria	Streptomyces griseus NBRC 13350	SGR_2214	<MglA<CvnC<MglB<HK	216
MglA Group 2	Actinobacteria	Streptomyces griseus NBRC 13350	SGR_1935	HK>MglB>CvnC>MglA>HK>MglB>CvnC>MglA>	174, 174
MglA Group 2	Actinobacteria	Streptomyces scabiei 87 22	SCAB_78801	HK>MglB>CvnC>MglA>	177
MglA Group 2	Actinobacteria	Streptomyces scabiei 87 22	SCAB_12711	<MglA<CvnC<MglB<HK	182
MglA Group 2	Actinobacteria	Streptomyces scabiei 87 22	SCAB_79171	HK>MglB>CvnC>MglA>	197
MglA Group 2	Actinobacteria	Streptomyces scabiei 87 22	SCAB_4651	<MglA<CvnC<MglB<HK	201
MglA Group 2	Actinobacteria	Streptomyces scabiei 87 22	SCAB_79951	HK>MglB>CvnC>MglA>MglB>	201
MglA Group 2	Actinobacteria	Streptomyces scabiei 87 22	SCAB_73681	HK>MglB>CvnC>MglA>	207
MglA Group 2	Actinobacteria	Streptomyces scabiei 87 22	SCAB_32081	HK>MglB>CvnC>MglA>	208
MglA Group 2	Actinobacteria	Streptomyces scabiei 87 22	SCAB_76101	HK>MglB>CvnC>MglA>	212
MglA Group 2	Actinobacteria	Streptomyces scabiei 87 22	SCAB_29671	<MglA<CvnC<MglB<HK	282
MglA Group 2	Actinobacteria	Streptomyces scabiei 87 22	SCAB_26691	HK>MglB>CvnC>MglA>HK>MglB>CvnC>MglA>	191, 193
MglA Group 2	Actinobacteria	Streptomyces scabiei 87 22	SCAB_20231	<MglA<CvnC<MglB<HK--HK>MglB>CvnC>MglA>--MglB>MglA>	210, 200, 200
MglA Group 2	Actinobacteria	Streptomyces SirexAA E	SACTE_2742	<MglA<CvnC<MglB<HK	188
MglA Group 2	Actinobacteria	Streptomyces SirexAA E	SACTE_0820	<MglA<CvnC<MglB<HK	201
MglA Group 2	Actinobacteria	Streptomyces SirexAA E	SACTE_5100	HK>MglB>CvnC>MglA>	204
MglA Group 2	Actinobacteria	Streptomyces SirexAA E	SACTE_5838	HK>MglB>CvnC>MglA>	204
MglA Group 2	Actinobacteria	Streptomyces SirexAA E	SACTE_5950	HK>MglB>CvnC>MglA>	206
MglA Group 2	Actinobacteria	Streptomyces SirexAA E	SACTE_4518	HK>MglB>CvnC>MglA>	216
MglA Group 2	Actinobacteria	Streptomyces SirexAA E	SACTE_3806	<MglA<CvnC<MglB<HK	218
MglA Group 2	Actinobacteria	Streptomyces SirexAA E	SACTE_4759	<MglA<CvnC<MglB<HK<MglA<CvnC<MglB<HK	193, 193
MglA Group 2	Actinobacteria	Streptomyces violaceusniger Tu 4113	Strvi_8505	HK>MglB>CvnC>MglA>	196
MglA Group 2	Actinobacteria	Streptomyces violaceusniger Tu 4113	Strvi_5711	<MglA<CvnC<MglB<HK	197
MglA Group 2	Actinobacteria	Streptomyces violaceusniger Tu 4113	Strvi_1215	HK>MglB>CvnC>MglA>	203
MglA Group 2	Actinobacteria	Streptomyces violaceusniger Tu 4113	Strvi_2191	HK>MglB>CvnC>MglA>	204
MglA Group 2	Actinobacteria	Streptomyces violaceusniger Tu 4113	Strvi_3550	HK>MglB>CvnC>MglA>	213
MglA Group 2	Actinobacteria	Streptomyces violaceusniger Tu 4113	Strvi_0346	HK>MglB>CvnC>MglA>	214
MglA Group 2	Actinobacteria	Streptomyces violaceusniger Tu 4113	Strvi_1624	HK>MglB>CvnC>MglA>	221
MglA Group 2	Actinobacteria	Streptomyces violaceusniger Tu 4113	Strvi_7653	HK>MglB>CvnC>MglA>	239
MglA Group 2	Actinobacteria	Streptomyces violaceusniger Tu 4113	Strvi_1890	<MglA<CvnC<MglB<HK<MglA<CvnC<MglB<HK	192, 194
MglA Group 2	Actinobacteria	Streptosporangium roseum DSM 43021	Sros_1808	HK>MglB>MglA>	179
MglA Group 2	Actinobacteria	Streptosporangium roseum DSM 43021	Sros_0356	HK>MglB>CvnC><MglA<CvnC<MglB	191
MglA Group 2	Actinobacteria	Streptosporangium roseum DSM 43021	Sros_1788	HK>MglB>CvnC>MglA>	195
MglA Group 2	Actinobacteria	Streptosporangium roseum DSM 43021	Sros_1821	HK>MglB>CvnC>MglA>	197
MglA Group 2	Actinobacteria	Streptosporangium roseum DSM 43021	Sros_3123	<MglA<CvnC<MglB<HK	199
MglA Group 2	Actinobacteria	Streptosporangium roseum DSM 43021	Sros_3193	HK>MglB>CvnC>MglA>	207
MglA Group 2	Actinobacteria	Thermobifida fusca YX	Tfu_2804	<MglA<CvnC<MglB<HK	188
MglA Group 2	Actinobacteria	Thermobifida fusca YX	Tfu_1430	<MglA<CvnC<MglB<HK	190
MglA Group 2	Actinobacteria	Thermobifida fusca YX	Tfu_0940	HK>MglB>CvnC>MglA>	195
MglA Group 2	Actinobacteria	Thermobifida fusca YX	Tfu_2444	HK>MglB>CvnC>MglA>	195
MglA Group 2	Actinobacteria	Thermobifida fusca YX	Tfu_0760	MglA>	196
MglA Group 2	Actinobacteria	Thermobifida fusca YX	Tfu_2044	<CvnC<HK-<MglA<CvnC<MglB<HK	197
MglA Group 2	Actinobacteria	Thermobispora bispora DSM 43833	Tbis_0153	<MglA<CvnC<MglB<HK	191
MglA Group 2	Actinobacteria	Thermobispora bispora DSM 43833	Tbis_3252	<MglA<CvnC<MglB<HK	195
MglA Group 2	Actinobacteria	Thermobispora bispora DSM 43833	Tbis_0970	HK>MglB>CvnC>MglA>HK>MglB>CvnC>MglA>	194, 208
MglA Group 2	Actinobacteria	Thermomonospora curvata DSM 43183	Tcur_1803	HK>MglB>CvnC>MglA>	192
MglA Group 2	Actinobacteria	Thermomonospora curvata DSM 43183	Tcur_3337	HK>MglB>CvnC>MglA>	193
MglA Group 2	Actinobacteria	Thermomonospora curvata DSM 43183	Tcur_2803	<MglA<CvnC<MglB<HK	204
MglA Group 2	Actinobacteria	Thermomonospora curvata DSM 43183	Tcur_3666	<MglA<CvnC<MglB<HK	205
MglA Group 2	Actinobacteria	Thermomonospora curvata DSM 43183	Tcur_1434	<MglA<CvnC<MglB<HK	213
MglA Group 2	Actinobacteria	Thermomonospora curvata DSM 43183	Tcur_3802	<MglA<CvnC<MglB<HK---MglB>CvnC>MglA>	202, 197
MglA Group 2	Actinobacteria	Verrucosispora maris AB 18 032	VAB18032_07335	HK>MglB>MglA>	179
MglA Group 2	Actinobacteria	Verrucosispora maris AB 18 032	VAB18032_08655	HK>MglB>CvnC>MglA>	197
MglA Group 2	Actinobacteria	Verrucosispora maris AB 18 032	VAB18032_25785	<MglA<CvnC<MglB<HK	200
MglA Group 2	Actinobacteria	Verrucosispora maris AB 18 032	VAB18032_29331	<MglA<CvnC<MglB<HK	200
MglA Group 2	Actinobacteria	Verrucosispora maris AB 18 032	VAB18032_00880	<MglA<CvnC<MglB<HK	207
MglA Group 2	Actinobacteria	Verrucosispora maris AB 18 032	VAB18032_02330	HK>MglB>CvnC>MglA>	250
MglA Group 2	Actinobacteria	Xylanimonas cellulosilytica DSM 15894	Xcel_0171	HK>MglB>CvnC>MglA>	310
MglA Group 3	Actinobacteria	Frankia EuI1c	FraEuI1c_3213	<MglA<DUF<MglBMglB>	198
MglA Group 3	Proteobacteria-beta	Collimonas fungivorans Ter331	CFU_2635	DUF>MglA>MglB>	189
MglA Group 3	Proteobacteria-beta	Comamonas testosteroni CNB 2	CtCNB1_0401	DUF>MglA>MglB>	188
MglA Group 3	Proteobacteria-beta	Delftia acidovorans SPH 1	Daci_0776	<MglA<DUF	182
MglA Group 3	Proteobacteria-beta	Delftia acidovorans SPH 1	Daci_0555	DUF>MglA>MglB>	187
MglA Group 3	Proteobacteria-beta	Delftia Cs1 4	DelCs14_5759	DUF>MglA>	182
MglA Group 3	Proteobacteria-beta	Delftia Cs1 4	DelCs14_0499	DUF>MglA>MglB>	187
MglA Group 3	Proteobacteria-beta	Leptothrix cholodnii SP 6	Lcho_0655	DUF>MglA>MglB>MglB>	178
MglA Group 3	Proteobacteria-beta	Leptothrix cholodnii SP 6	Lcho_2393	DUF>MglA>	207
MglA Group 3	Proteobacteria-beta	Neisseria lactamica 020 06	NLA_5090	DUF>MglA>MglB>MglB>	178
MglA Group 3	Proteobacteria-beta	Neisseria meningitidis 053442	NMCC_1663	<MglB<MglB<MglA<DUF	178
MglA Group 3	Proteobacteria-beta	Neisseria meningitidis alpha14	NMO_1563	<MglB<MglB<MglA<DUF	178
MglA Group 3	Proteobacteria-beta	Neisseria meningitidis FAM18	NMC1669	<MglB<MglB<MglA<DUF	178
MglA Group 3	Proteobacteria-beta	Neisseria meningitidis MC58	NMB0476	DUF>MglA>MglB>MglB>	178
MglA Group 3	Proteobacteria-beta	Neisseria meningitidis Z2491	NMA2006	<MglB<MglB<MglA<DUF	178
MglA Group 3	Proteobacteria-beta	Polaromonas naphthalenivorans CJ2	Pnap_0367	<MglB--<MglA<DUF	187
MglA Group 3	Proteobacteria-beta	Variovorax paradoxus EPS	Varpa_3016	DUF>MglA>MglB>	172
MglA Group 3	Proteobacteria-beta	Variovorax paradoxus EPS	Varpa_4909	<MglB<MglA<DUF	180
MglA Group 3	Proteobacteria-gamma	Acidithiobacillus caldus SM 1	Atc_2384	DUF>MglA>MglB>MglB>	188
MglA Group 3	Proteobacteria-gamma	Acidithiobacillus caldus SM 1	Atc_2681	<MglA	188
MglA Group 3	Proteobacteria-gamma	Acidithiobacillus ferrivorans SS3	Acife_2509	DUF>MglA>MglB>MglB>	188
MglA Group 3	Proteobacteria-gamma	Acidithiobacillus ferrooxidans ATCC 23270	AFE_2575	DUF>MglA>MglB>MglB>	188
MglA Group 3	Proteobacteria-gamma	Acidithiobacillus ferrooxidans ATCC 53993	Lferr_2205	DUF>MglA>MglB>MglB>	188
MglA Group 3	Proteobacteria-gamma	Acinetobacter calcoaceticus PHEA 2	BDGL_002466	DUF>MglA>MglB>	186
MglA Group 3	Proteobacteria-gamma	Acinetobacter oleivorans DR1	AOLE_02455	<MglB<MglA<DUF	186
MglA Group 3	Proteobacteria-gamma	Citrobacter rodentium ICC168	ROD_41482	DUF>MglA>MglB>	186
MglA Group 3	Proteobacteria-gamma	Dichelobacter nodosus VCS1703A	DNO_0145	<MglB<MglA<DUF	176
MglA Group 3	Proteobacteria-gamma	Methylomicrobium alcaliphilum	MEALZ_2464	<MglB<MglB<MglA<DUF	178
MglA Group 3	Proteobacteria-gamma	Methylomonas methanica MC09	Metme_1347	DUF>MglA>MglB>MglB>	179
MglA Group 3	Proteobacteria-gamma	Nitrosococcus halophilus Nc4	Nhal_3340	<MglB<MglA<DUF	181
MglA Group 3	Proteobacteria-gamma	Nitrosococcus oceani ATCC 19707	Noc_0132	DUF>MglA>MglB>	181
MglA Group 3	Proteobacteria-gamma	Nitrosococcus watsonii C 113	Nwat_0121	DUF>MglA>	181
MglA Group 3	Proteobacteria-gamma	Stenotrophomonas maltophilia JV3	BurJV3_2798	DUF>MglA>MglB>	183
MglA Group 3	Proteobacteria-gamma	Stenotrophomonas maltophilia JV3	BurJV3_3715	<MglB<MglA<DUF	183
MglA Group 3	Proteobacteria-gamma	Stenotrophomonas maltophilia K279a	Smlt4270	<MglB<MglA<DUF	182
MglA Group 3	Proteobacteria-gamma	Stenotrophomonas maltophilia R551 3	Smal_3681	<MglB<MglA<DUF	182
MglA Group 3	Proteobacteria-gamma	Teredinibacter turnerae T7901	TERTU_3407	<MglB<MglA<DUF	175
MglA Group 3	Proteobacteria-gamma	Teredinibacter turnerae T7901	TERTU_4498	DUF>MglA>MglB>MglB>	178
MglA Group 3	Proteobacteria-gamma	Xanthomonas axonopodis citri 306	XAC4010	MglB>-DUF>MglA>	180
MglA Group 3	Proteobacteria-gamma	Xanthomonas axonopodis citrumelo F1	XACM_3879	MglB>-DUF>MglA>	180

MglA Group 3	Proteobacteria-gamma	Xanthomonas campestris 8004	XC_4016	MglB>-DUF>MglA>	180
MglA Group 3	Proteobacteria-gamma	Xanthomonas campestris ATCC 33913	XCC3927	MglB>-DUF>MglA>	180
MglA Group 3	Proteobacteria-gamma	Xanthomonas campestris B100	xccb100_4114	MglB>-DUF>MglA>	180
MglA Group 3	Proteobacteria-gamma	Xanthomonas campestris vesicatoria 85 10	XCV4103	MglB>-MglA>	444
MglA Group 4	Euryarchaeota	Archaeoglobus veneficus SNP6	Arcve_1631	MglA>MglB>	385
MglA Group 4	Euryarchaeota	Methanobacterium AL 21	Metbo_0941	<MglA	157
MglA Group 4	Euryarchaeota	Methanobacterium AL 21	Metbo_2322	<MglA	305
MglA Group 4	Euryarchaeota	Methanobacterium AL 21	Metbo_1335	MglB>MglA>	390
MglA Group 4	Euryarchaeota	Methanobacterium SWAN 1	MSWAN_1306	MglA>	158
MglA Group 4	Euryarchaeota	Methanobacterium SWAN 1	MSWAN_1484	MglB>-MglB>-MglA>MglB>MglB>	169
MglA Group 4	Euryarchaeota	Methanobacterium SWAN 1	MSWAN_1797	MglA>--MglA>	178, 303
MglA Group 4	Euryarchaeota	Methanocaldococcus fervens AG86	Mefer_0171	MglA>MglB>	153
MglA Group 4	Euryarchaeota	Methanocaldococcus FS406 22	MFS40622_0221	MglA>MglB>	157
MglA Group 4	Euryarchaeota	Methanocaldococcus jannaschii DSM 2661	MJ_1339	MglA>MglB>	154
MglA Group 4	Euryarchaeota	Methanocaldococcus vulcanius M7	Metvu_1719	MglA>MglB>	156
MglA Group 4	Euryarchaeota	Methanocella paludicola SANAE	MCP_2943	<MglA	171
MglA Group 4	Euryarchaeota	Methanocella paludicola SANAE	MCP_0104	<MglA:MglB	523
MglA Group 4	Euryarchaeota	Methanococcus maripaludis C5	MmarC5_1691	MglA>MglB>	157
MglA Group 4	Euryarchaeota	Methanococcus maripaludis C6	MmarC6_0956	MglA>MglB>	157
MglA Group 4	Euryarchaeota	Methanococcus maripaludis C7	MmarC7_0989	<MglB<MglA	157
MglA Group 4	Euryarchaeota	Methanococcus maripaludis S2	MMP1714	<MglB<MglA	157
MglA Group 4	Euryarchaeota	Methanococcus maripaludis X1	GYY_09445	<MglB<MglA	157
MglA Group 4	Euryarchaeota	Methanococcus vannielii SB	Mevan_1016	<MglB<MglA	158
MglA Group 4	Euryarchaeota	Methanoculleus marisnigri JR1	Memar_2190	<MglA	161
MglA Group 4	Euryarchaeota	Methanopyrus kandleri AV19	MK1372	MglA>	187
MglA Group 4	Euryarchaeota	Methanoregula boonei 6A8	Mboo_0457	MglA>	168
MglA Group 4	Euryarchaeota	Methanoregula boonei 6A8	Mboo_2067	MglA>	387
MglA Group 4	Euryarchaeota	Methanothermobacter marburgensis Marburg	MTBMA_c11600	<MglA	151
MglA Group 4	Euryarchaeota	Methanothermobacter marburgensis Marburg	MTBMA_c09740	<MglA<MglB	391
MglA Group 4	Euryarchaeota	Methanothermobacter thermautotrophicus Delta H	MTH765	<MglA	152
MglA Group 4	Euryarchaeota	Methanothermobacter thermautotrophicus Delta H	MTH594	<MglA<MglB	393
MglA Group 4	Euryarchaeota	Methanothermococcus okinawensis IH1	Metok_0410	MglA>MglB>	163
MglA Group 4	Euryarchaeota	Methanotorris igneus Kol 5	Metig_0996	<MglB<MglA	162
MglA Group 4	Euryarchaeota	uncultured methanogenic archaeon RC I	RCIX894	MglA>	174
MglA Group 4	Euryarchaeota	uncultured methanogenic archaeon RC I	RCIX296	<MglB<MglA	401
MglA Group 4	Korarchaeota	Candidatus Korarchaeum cryptofilum OPF8	Kcr_0903	<MglA	201
MglA Group 5	Actinobacteria	Acidothermus cellulolyticus 11B	Acel_1323	<MglA<PATAN<MglB	193
MglA Group 5	Actinobacteria	Kineococcus radiotolerans SRS30216	Krad_3042	MglA>MglB>	200
MglA Group 5	Actinobacteria	Sanguibacter keddieii DSM 10542	Sked_01150	<MglB<MglA	191
MglA Group 5	Aquificae	Aquifex aeolicus VF5	aq_1844	<MglA<PATAN<MglB	186
MglA Group 5	Aquificae	Desulfurobacterium thermolithotrophum DSM 11699	Dester_1501	MglA>---MglB>	175
MglA Group 5	Aquificae	Hydrogenobacter thermophilus TK 6	HTH_0572	MglB>PATAN>MglA>	182
MglA Group 5	Aquificae	Persephonella marina EX H1	PERMA_1733	MglB>PATAN>MglA>	180
MglA Group 5	Aquificae	Sulfurihydrogenibium azorense Az Fu1	SULAZ_1509	MglB>PATAN>MglA>	177
MglA Group 5	Aquificae	Sulfurihydrogenibium YO3AOP1	SYO3AOP1_0078	MglB>PATAN>MglA>	177
MglA Group 5	Aquificae	Thermocrinis albus DSM 14484	Thal_0484	MglB>PATAN>MglA>	182
MglA Group 5	Chlorobi	Chloroherpeton thalassium ATCC 35110	Ctha_0548	MglB>-MglA>	178
MglA Group 5	Chlorobi	Chloroherpeton thalassium ATCC 35110	Ctha_0677	<MglA--<MglB	183
MglA Group 5	Chloroflexi	Anaerolinea thermophila UNI 1	ANT_06780	<MglA<PATAN	179
MglA Group 5	Chloroflexi	Chloroflexus aggregans DSM 9485	Cagg_2798	PATAN>MglA>	176
MglA Group 5	Chloroflexi	Chloroflexus aurantiacus J 10 fl	Caur_0665	<MglA<PATAN	176
MglA Group 5	Chloroflexi	Chloroflexus Y 400 fl	Chy400_0720	<MglA<PATAN	176
MglA Group 5	Chloroflexi	Herpetosiphon aurantiacus DSM 785	Haur_4502	MglA>	172
MglA Group 5	Chloroflexi	Herpetosiphon aurantiacus DSM 785	Haur_0845	PATAN>MglA>	176
MglA Group 5	Chloroflexi	Herpetosiphon aurantiacus DSM 785	Haur_0378	PATAN>MglA>MglB>	191
MglA Group 5	Chloroflexi	Roseiflexus castenholzii DSM 13941	Rcas_1730	<MglA<PATAN	176
MglA Group 5	Chloroflexi	Roseiflexus castenholzii DSM 13941	Rcas_3230	<MglA<PATAN-<MglB	177
MglA Group 5	Chloroflexi	Roseiflexus RS 1	RoseRS_2140	PATAN>MglA>	176
MglA Group 5	Chloroflexi	Roseiflexus RS 1	RoseRS_3912	MglB>-PATAN>MglA>	177
MglA Group 5	Crenarchaeota	Caldivirga maquilingensis IC 167	Cmaq_1262	<MglA	187
MglA Group 5	Crenarchaeota	Thermofilum pendens Hrk 5	Tpen_0253	MglA>MglB>	182
MglA Group 5	Crenarchaeota	Vulcanisaeta distributa DSM 14429	Vdis_1195	<MglA	184
MglA Group 5	Crenarchaeota	Vulcanisaeta moutnovskia 768 28	VMUT_2043	<MglA	187
MglA Group 5	Cyanobacteria	Cyanothece PCC 7425	Cyan7425_5249	<MglB<MglA<PATAN	180
MglA Group 5	Cyanobacteria	Cyanothece PCC 7425	Cyan7425_2399	<MglB-<MglA<PATAN	185
MglA Group 5	Cyanobacteria	Synechococcus JA 2 3B a 2 13	CYB_1482	PATAN>PATAN>MglB>-MglA>	181
MglA Group 5	Cyanobacteria	Synechococcus JA 3 3Ab	CYA_2865	<MglA-<MglB<PATAN<PATAN<PATAN	179
MglA Group 5	Cyanobacteria	Thermosynechococcus elongatus BP 1	tlr1990	MglB>MglA>PATAN>	188
MglA Group 5	Deinococcus-Thermus	Deinococcus maricopensis DSM 21211	Deima_0315	PATAN>MglA>MglB>	174
MglA Group 5	Deinococcus-Thermus	Deinococcus proteolyticus MRP	Deipr_2135	PATAN>MglA>MglB>	184
MglA Group 5	Deinococcus-Thermus	Deinococcus radiodurans R1	DR_2180	<MglB<MglA<PATAN	184
MglA Group 5	Deinococcus-Thermus	Oceanithermus profundus DSM 14977	Ocepr_0168	PATAN>MglA>MglB>	167
MglA Group 5	Proteobacteria-delta	Hippea maritima DSM 10411	Hipma_1035	MglA>	182
MglA Group 5	Verrucomicrobia	Methylacidiphilum infernorum V4	Minf_2415	MglA>MglB>	174
Roco	Actinobacteria	Streptomyces scabiei 87 22	SCAB_35141	Roco>	743
Roco	Bacteroidetes	Haliscomenobacter hydrossis DSM 1100	Halhy_4925	Roco>	840
Roco	Bacteroidetes	Haliscomenobacter hydrossis DSM 1100	Halhy_0082	<Roco	995
Roco	Bacteroidetes	Haliscomenobacter hydrossis DSM 1100	Halhy_6078	<Roco	998
Roco	Bacteroidetes	Haliscomenobacter hydrossis DSM 1100	Halhy_6692	<Roco	1023
Roco	Bacteroidetes	Haliscomenobacter hydrossis DSM 1100	Halhy_1222	<Roco	1108
Roco	Bacteroidetes	Haliscomenobacter hydrossis DSM 1100	Halhy_6666	<Roco	1141
Roco	Bacteroidetes	Haliscomenobacter hydrossis DSM 1100	Halhy_4373	<Roco	1448
Roco	Bacteroidetes	Haliscomenobacter hydrossis DSM 1100	Halhy_6541	<Roco<Roco<Roco	741, 786, 1058
Roco	Bacteroidetes	Porphyromonas gingivalis ATCC 33277	PGN_1796	<Roco	1125
Roco	Bacteroidetes	Porphyromonas gingivalis TDC60	PGTDC60_0128	<Roco	1384
Roco	Bacteroidetes	Porphyromonas gingivalis W83	PG1864	<Roco	1266
Roco	Bacteroidetes	Spirosoma linguale DSM 74	Slin_6989	Roco>	925
Roco	Chlorobi	Chlorobium chlorochromatii CaD3	Cag_0507	Roco>Roco>	614, 811
Roco	Chlorobi	Chlorobium limicola DSM 245	Clim_1476	Roco>	998
Roco	Chlorobi	Chlorobium tepidum TLS	CT1526	Roco>	1102
Roco	Chlorobi	Chloroherpeton thalassium ATCC 35110	Ctha_0692	<Roco	394
Roco	Cyanobacteria	Acaryochloris marina MBIC11017	AM1_4747	Roco>	705
Roco	Cyanobacteria	Acaryochloris marina MBIC11017	AM1_1973	Roco>	842
Roco	Cyanobacteria	Acaryochloris marina MBIC11017	AM1_2164	Roco>	1409
Roco	Cyanobacteria	Anabaena variabilis ATCC 29413	Ava_1493	Roco>	1107
Roco	Cyanobacteria	Cyanothece PCC 7424	PCC7424_3253	Roco>	867
Roco	Cyanobacteria	Cyanothece PCC 7424	PCC7424_4909	Roco>	1015
Roco	Cyanobacteria	Cyanothece PCC 7822	Cyan7822_3827	<Roco	857
Roco	Cyanobacteria	Cyanothece PCC 7822	Cyan7822_0792	Roco>	876
Roco	Cyanobacteria	Cyanothece PCC 8801	PCC8801_3999	<Roco	937
Roco	Cyanobacteria	Cyanothece PCC 8802	Cyan8802_4041	<Roco	937

Roco	Cyanobacteria	Nostoc PCC 7120	alr0124	Roco>	1119
Roco	Cyanobacteria	Nostoc punctiforme PCC 73102	Npun_F6039	Roco>	1109
Roco	Cyanobacteria	Nostoc punctiforme PCC 73102	Npun_R6524	<Roco	1124
Roco	Cyanobacteria	Nostoc punctiforme PCC 73102	Npun_F1213	Roco>	1185
Roco	Cyanobacteria	Trichodesmium erythraeum IMS101	Tery_1428	<Roco	748
Roco	Cyanobacteria	Trichodesmium erythraeum IMS101	Tery_3798	Roco>	1041
Roco	Euryarchaeota	Methanosarcina acetivorans C2A	MA2301	Roco>	631
Roco	Euryarchaeota	Methanosarcina barkeri Fusaro	Mbar_A2306	Roco>	863
Roco	Euryarchaeota	Methanosarcina barkeri Fusaro	Mbar_A2449	Roco>	886
Roco	Euryarchaeota	Methanosarcina barkeri Fusaro	Mbar_A3344	<Roco	892
Roco	Proteobacteria-alpha	Azospirillum lipoferum 4B	AZOLI_2723	<Roco	1026
Roco	Proteobacteria-alpha	Rhodospirillum rubrum ATCC 11170	Rru_A0837	<Roco	1085
Roco	Proteobacteria-delta	Desulfatibacillum alkenivorans AK 01	Dalk_3770	Roco>	792
Roco	Proteobacteria-gamma	Methylomonas methanica MC09	Metme_1781	<Roco	944
Roco	Unclassified	Magnetococcus MC 1	Mmc1_0461	<Roco	761
Rup Group 1	Bacteroidetes	Cellulophaga algicola DSM 14237	Celal_1350	<Rup<DUFX<DUFY	162
Rup Group 1	Bacteroidetes	Croceibacter atlanticus HTCC2559	CA2559_02285	<Rup<DUFX<DUFY	161
Rup Group 1	Bacteroidetes	Flavobacteriales bacterium HTCC2170	FB2170_04570	<Rup<DUFX<DUFY	165
Rup Group 1	Bacteroidetes	Flavobacterium branchiophilum FL 15	FBFL15_2718	DUFY>DUFX>Rup>	163
Rup Group 1	Bacteroidetes	Krokinobacter 4H 3 7 5	Krodi_0967	<Rup<DUFX<DUFY	164
Rup Group 1	Bacteroidetes	Lacinutrix 5H 3 7 4	Lacal_0783	DUFY>DUFX>Rup>	163
Rup Group 1	Chloroflexi	Chloroflexus aggregans DSM 9485	Cagg_2203	Rup>DUFY>DUFX>	176
Rup Group 1	Chloroflexi	Chloroflexus aurantiacus J 10 fl	Caur_1236	Rup>DUFY>DUFX>	172
Rup Group 1	Chloroflexi	Chloroflexus Y 400 fl	Chy400_1355	Rup>DUFY>DUFX>	172
Rup Group 1	Chloroflexi	Roseiflexus castenholzii DSM 13941	Rcas_3601	<DUFX<DUFY<Rup	174
Rup Group 1	Cyanobacteria	Acaryochloris marina MBIC11017	AM1_6349	Rup>	160
Rup Group 1	Cyanobacteria	Acaryochloris marina MBIC11017	AM1_2998	<Rup<DUFX:OmpA<DUFY	163
Rup Group 1	Cyanobacteria	Anabaena variabilis ATCC 29413	Ava_0879	DUFY>DUFX:OmpA>Rup>	165
Rup Group 1	Cyanobacteria	Cyanothece ATCC 51142	cce_4705	DUFY>-OmpA:DUFX>Rup>	181
Rup Group 1	Cyanobacteria	Nostoc PCC 7120	all3030	<Rup<OmpA:DUFX<DUFY	165
Rup Group 1	Cyanobacteria	Nostoc punctiforme PCC 73102	Npun_F1891	DUFY>OmpA:DUFX>Rup>	166
Rup Group 1	Nitrospirae	Candidatus Nitrospira defluvii	NIDE0273	<Rup<DUFX:OmpA<DUFY	178
Rup Group 1	Proteobacteria-alpha	Mesorhizobium loti MAFF303099	mll3243	<Rup<OmpA:DUFX<DUFY	168
Rup Group 1	Proteobacteria-delta	Sorangium cellulosum So ce 56	sce5816	<Rup<OmpA:DUFX<DUFY	173
Rup Group 1	Proteobacteria-epsilon	Sulfurovum NBC37 1	SUN_0835	DUFY>OmpA:DUFX>Rup>	158
Rup Group 1	Proteobacteria-gamma	Alteromonas macleodii Deep ecotype	MADE_1016925	DUFY>DUFX:OmpA>Rup>	161
Rup Group 1	Proteobacteria-gamma	Alteromonas macleodii Deep ecotype	MADE_1017110	<Rup<DUFX:OmpA<DUFY	161
Rup Group 1	Proteobacteria-gamma	Alteromonas SN2	ambt_00690	<Rup<DUFX:OmpA<DUFY	161
Rup Group 1	Proteobacteria-gamma	Glaciecola 4H 3 7 YE 5	Glaag_0299	<Rup<OmpA:DUFX<DUFY	167
Rup Group 1	Proteobacteria-gamma	Glaciecola nitratireducens FR1064	GNIT_3134	DUFY>OmpA:DUFX>Rup>	164
Rup Group 1	Proteobacteria-gamma	Marinomonas mediterranea MMB 1	Marme_1218	DUFY>OmpA:DUFX>Rup>	164
Rup Group 1	Proteobacteria-gamma	Pseudoalteromonas atlantica T6c	Patl_3989	DUFY>OmpA:DUFX>Rup>	167
Rup Group 2	Crenarchaeota	Pyrobaculum 1860	P186_1425	Rup>	152
Rup Group 2	Crenarchaeota	Pyrobaculum 1860	P186_1746	Rup>	156
Rup Group 2	Crenarchaeota	Pyrobaculum 1860	P186_0647	Rup>	161
Rup Group 2	Crenarchaeota	Pyrobaculum 1860	P186_1318	<Rup	162
Rup Group 2	Crenarchaeota	Pyrobaculum 1860	P186_1238	<Rup	166
Rup Group 2	Crenarchaeota	Pyrobaculum aerophilum IM2	PAE2789	<Rup	156
Rup Group 2	Crenarchaeota	Pyrobaculum aerophilum IM2	PAE3253	Rup>	158
Rup Group 2	Crenarchaeota	Pyrobaculum aerophilum IM2	PAE3403	Rup>	160
Rup Group 2	Crenarchaeota	Pyrobaculum aerophilum IM2	PAE3492	Rup>	161
Rup Group 2	Crenarchaeota	Pyrobaculum aerophilum IM2	PAE3290	Rup>	162
Rup Group 2	Crenarchaeota	Pyrobaculum aerophilum IM2	PAE1958	Rup>	169
Rup Group 2	Crenarchaeota	Pyrobaculum arsenaticum DSM 13514	Pars_0860	<Rup	156
Rup Group 2	Crenarchaeota	Pyrobaculum arsenaticum DSM 13514	Pars_1801	<Rup	158
Rup Group 2	Crenarchaeota	Pyrobaculum arsenaticum DSM 13514	Pars_1967	<Rup	161
Rup Group 2	Crenarchaeota	Pyrobaculum arsenaticum DSM 13514	Pars_2034	Rup>	163
Rup Group 2	Crenarchaeota	Pyrobaculum arsenaticum DSM 13514	Pars_1716	Rup>	164
Rup Group 2	Crenarchaeota	Pyrobaculum calidifontis JCM 11548	Pcal_0171	Rup>	162
Rup Group 2	Crenarchaeota	Pyrobaculum calidifontis JCM 11548	Pcal_1168	<Rup	162
Rup Group 2	Crenarchaeota	Pyrobaculum calidifontis JCM 11548	Pcal_0906	<Rup	165
Rup Group 2	Crenarchaeota	Pyrobaculum calidifontis JCM 11548	Pcal_1761	<Rup	170
Rup Group 2	Crenarchaeota	Pyrobaculum calidifontis JCM 11548	Pcal_1851	<Rup	171
Rup Group 2	Crenarchaeota	Pyrobaculum islandicum DSM 4184	Pisl_0786	Rup>	161
Rup Group 2	Crenarchaeota	Pyrobaculum islandicum DSM 4184	Pisl_1602	<Rup	164
Rup Group 2	Crenarchaeota	Pyrobaculum oguniense TE7	Pogu_1480	Rup>	156
Rup Group 2	Crenarchaeota	Pyrobaculum oguniense TE7	Pogu_0092	<Rup	158
Rup Group 2	Crenarchaeota	Pyrobaculum oguniense TE7	Pogu_0330	Rup>	158
Rup Group 2	Crenarchaeota	Pyrobaculum oguniense TE7	Pogu_0418	<Rup	177
Rup Group 2	Crenarchaeota	Thermoproteus neutrophilus V24Sta	Tneu_1638	Rup>	152
Rup Group 2	Crenarchaeota	Thermoproteus neutrophilus V24Sta	Tneu_0864	Rup>	161
Rup Group 2	Crenarchaeota	Thermoproteus neutrophilus V24Sta	Tneu_1252	Rup>	165
Rup Group 2	Crenarchaeota	Thermoproteus neutrophilus V24Sta	Tneu_0632	<Rup	167
Rup Group 2	Crenarchaeota	Thermoproteus neutrophilus V24Sta	Tneu_1536	Rup>	168
Rup Group 2	Crenarchaeota	Thermoproteus tenax Kra 1	TTX_0725	Rup>	161
Rup Group 2	Crenarchaeota	Thermoproteus uzoniensis 768 20	TUZN_0374	Rup>	158
Rup Unc	Bacteroidetes	Saprospira grandis Lewin	SGRA_3813	<Rup	151
Rup Unc	Chloroflexi	Sphaerobacter thermophilus DSM 20745	Sthe_1175	Rup>	167
Rup Unc	Crenarchaeota	Thermofilum pendens Hrk 5	Tpen_0080	<Rup	171
Rup Unc	Euryarchaeota	Aciduliprofundum boonei T469	Aboo_0571	Rup>	157
Rup Unc	Euryarchaeota	Thermoplasma acidophilum DSM 1728	Ta1192	<Rup	168
Rup Unc	Euryarchaeota	Thermoplasma volcanium GSS1	TVN0401	<Rup	171
Rup Unc	Korarchaeota	Candidatus Korarchaeum cryptofilum OPF8	Kcr_0908	Rup>	300
Rup Unc	Proteobacteria-alpha	Rhodomicrobium vannielii ATCC 17100	Rvan_3466	<Rup	171
Rup Unc	Proteobacteria-delta	Sorangium cellulosum So ce 56	sce1613	<Rup	339
Rup Unc	Proteobacteria-gamma	Acinetobacter baumannii AB307 0294	ABBFA_000211	Rup>	230
Rup Unc	Proteobacteria-gamma	Legionella longbeachae NSW150	LLO_2424	<Rup	175
Rup Unc	Proteobacteria-gamma	Legionella longbeachae NSW150	LLO_2249	Rup>	176
Rup Unc	Proteobacteria-gamma	Legionella longbeachae NSW150	LLO_3288	<Rup	306
Rup Unc	Proteobacteria-gamma	Legionella longbeachae NSW150	LLO_2329	Rup>	330
Rup Unc	Proteobacteria-gamma	Legionella longbeachae NSW150	LLO_1716	<Rup	332

Table S3. Orphan MglB sequences. Taxonomy, genome, locus, and gene neighborhood data are shown. The locus corresponds to the first sequence in the gene neighborhood.

Table S3. Orphan MglB sequences. Taxonomy, genome, locus, and gene neighborhood data are shown. The locus corresponds to the first sequence in the gene neighborhood.

Taxonomy	Genome	Locus	Gene neighborhood
Actinobacteria	Actinosynnema mirum DSM 43827	Amir_3807	<MglB
Actinobacteria	Actinosynnema mirum DSM 43827	Amir_5171	<MglB
Actinobacteria	Amycolatopsis mediterranei U32	AMED_3175	MglB>
Actinobacteria	Amycolatopsis mediterranei U32	AMED_3309	MglB>
Actinobacteria	Amycolatopsis mediterranei U32	AMED_7882	MglB>
Actinobacteria	Catenulispora acidiphila DSM 44928	Caci_3382	MglB>
Actinobacteria	Catenulispora acidiphila DSM 44928	Caci_4122	<MglB
Actinobacteria	Frankia alni ACN14a	FRAAL0016	MglB>
Actinobacteria	Frankia alni ACN14a	FRAAL4352	<MglB
Actinobacteria	Frankia CcI3	Francci3_2825	<MglB
Actinobacteria	Frankia EAN1pec	Franean1_2277	<MglB
Actinobacteria	Frankia EuI1c	FraEuI1c_5079	<MglB
Actinobacteria	Isoptericola variabilis 225	Isova_2837	<MglB
Actinobacteria	Kitasatospora setae KM 6054	KSE_67090	<MglB
Actinobacteria	Micromonospora aurantiaca ATCC 27029	Micau_4169	<MglB
Actinobacteria	Micromonospora aurantiaca ATCC 27029	Micau_4646	MglB>
Actinobacteria	Micromonospora aurantiaca ATCC 27029	Micau_4795	MglB>
Actinobacteria	Micromonospora aurantiaca ATCC 27029	Micau_5763	MglB>
Actinobacteria	Micromonospora L5	ML5_2732	<MglB
Actinobacteria	Micromonospora L5	ML5_3504	<MglB
Actinobacteria	Micromonospora L5	ML5_3655	<MglB
Actinobacteria	Micromonospora L5	ML5_4133	MglB>
Actinobacteria	Nocardia farcinica IFM 10152	nfa9800	<MglB
Actinobacteria	Nocardia farcinica IFM 10152	nfa47710	<MglB
Actinobacteria	Nocardiopsis dassonvillei DSM 43111	Ndas_1599	MglB>
Actinobacteria	Nocardiopsis dassonvillei DSM 43111	Ndas_3273	MglB>
Actinobacteria	Rhodococcus jostii RHA1	RHA1_ro03676	MglB>
Actinobacteria	Rubrobacter xylanophilus DSM 9941	Rxyl_2217	<MglB
Actinobacteria	Salinispora arenicola CNS 205	Sare_0700	MglB>
Actinobacteria	Salinispora tropica CNB 440	Strop_0758	MglB>
Actinobacteria	Stackebrandtia nassauensis DSM 44728	Snas_0738	<MglB
Actinobacteria	Stackebrandtia nassauensis DSM 44728	Snas_1515	MglB>
Actinobacteria	Stackebrandtia nassauensis DSM 44728	Snas_2845	MglB>
Actinobacteria	Stackebrandtia nassauensis DSM 44728	Snas_3691	<MglB
Actinobacteria	Stackebrandtia nassauensis DSM 44728	Snas_4131	MglB>
Actinobacteria	Stackebrandtia nassauensis DSM 44728	Snas_5598	<MglB
Actinobacteria	Streptomyces avermitilis MA 4680	SAV_3500	MglB>
Actinobacteria	Streptomyces avermitilis MA 4680	SAV_6890	MglB>
Actinobacteria	Streptomyces bingchenggensis BCW 1	SBI_01696	<MglB--<MglB
Actinobacteria	Streptomyces bingchenggensis BCW 1	SBI_01847	MglB>
Actinobacteria	Streptomyces bingchenggensis BCW 1	SBI_02628	MglB>
Actinobacteria	Streptomyces bingchenggensis BCW 1	SBI_07280	<MglB
Actinobacteria	Streptomyces bingchenggensis BCW 1	SBI_07328	<MglB
Actinobacteria	Streptomyces cattleya NRRL 8057	SCAT_0875	<MglB
Actinobacteria	Streptomyces cattleya NRRL 8057	SCAT_p1057	MglB>
Actinobacteria	Streptomyces coelicolor A3 2	SCO0683	<MglB
Actinobacteria	Streptomyces flavogriseus ATCC 33331	Sfla_0168	<MglB
Actinobacteria	Streptomyces flavogriseus ATCC 33331	Sfla_3832	<MglB
Actinobacteria	Streptomyces griseus NBRC 13350	SGR_335	<MglB
Actinobacteria	Streptomyces griseus NBRC 13350	SGR_1526	MglB>
Actinobacteria	Streptomyces griseus NBRC 13350	SGR_4458	<MglB
Actinobacteria	Streptomyces scabiei 87 22	SCAB_29691	<MglB
Actinobacteria	Streptomyces scabiei 87 22	SCAB_30321	<MglB
Actinobacteria	Streptomyces scabiei 87 22	SCAB_51131	<MglB
Actinobacteria	Streptomyces scabiei 87 22	SCAB_54661	<MglB
Actinobacteria	Streptomyces SirexAA E	SACTE_2536	MglB>

Actinobacteria	Streptomyces SirexAA E	SACTE_6441	MglB>
Actinobacteria	Streptomyces violaceusniger Tu 4113	Strvi_9310	<MglB
Actinobacteria	Streptomyces violaceusniger Tu 4113	Strvi_3289	MglB>
Actinobacteria	Streptomyces violaceusniger Tu 4113	Strvi_4113	MglB>--MglB>
Actinobacteria	Streptomyces violaceusniger Tu 4113	Strvi_5170	MglB>
Actinobacteria	Streptosporangium roseum DSM 43021	Sros_7712	MglB>
Actinobacteria	Thermobifida fusca YX	Tfu_2113	MglB>
Actinobacteria	Thermomonospora curvata DSM 43183	Tcur_0274	MglB>
Actinobacteria	Verrucosispora maris AB 18 032	VAB18032_24230	<MglB
Actinobacteria	Verrucosispora maris AB 18 032	VAB18032_25115	<MglB
Actinobacteria	Verrucosispora maris AB 18 032	VAB18032_27576	MglB>
Actinobacteria	Verrucosispora maris AB 18 032	VAB18032_28161	MglB>
Actinobacteria	Verrucosispora maris AB 18 032	VAB18032_02335	MglB>
Actinobacteria	Xylanimonas cellulosilytica DSM 15894	Xcel_0172	MglB>
Aquificae	Aquifex aeolicus VF5	aq_1822	MglB>
Aquificae	Sulfurihydrogenibium YO3AOP1	SYO3AOP1_1333	<MglB
Aquificae	Thermovibrio ammonificans HB 1	Theam_0105	MglB>
Aquificae	Thermovibrio ammonificans HB 1	Theam_1630	MglB>
Aquificae	Thermovibrio ammonificans HB 1	Theam_1718	MglB>
Bacteroidetes	Flavobacterium johnsoniae UW101	Fjoh_0695	<MglB
Bacteroidetes	Flavobacterium johnsoniae UW101	Fjoh_0701	MglB>
Chlorobi	Chloroherpeton thalassium ATCC 35110	Ctha_2351	<MglB<MglB
Chloroflexi	Anaerolinea thermophila UNI 1	ANT_03760	MglB>
Chloroflexi	Chloroflexus aggregans DSM 9485	Cagg_0992	<MglB
Chloroflexi	Chloroflexus aggregans DSM 9485	Cagg_2813	MglB>
Chloroflexi	Chloroflexus aggregans DSM 9485	Cagg_3071	MglB>
Chloroflexi	Chloroflexus aggregans DSM 9485	Cagg_3202	MglB>
Chloroflexi	Chloroflexus aggregans DSM 9485	Cagg_3625	MglB>
Chloroflexi	Chloroflexus aurantiacus J 10 fl	Caur_0276	<MglB
Chloroflexi	Chloroflexus aurantiacus J 10 fl	Caur_0520	<MglB
Chloroflexi	Chloroflexus aurantiacus J 10 fl	Caur_0696	MglB>
Chloroflexi	Chloroflexus aurantiacus J 10 fl	Caur_2319	<MglB
Chloroflexi	Chloroflexus aurantiacus J 10 fl	Caur_3048	<MglB
Chloroflexi	Chloroflexus Y 400 fl	Chy400_0296	<MglB
Chloroflexi	Chloroflexus Y 400 fl	Chy400_0556	<MglB
Chloroflexi	Chloroflexus Y 400 fl	Chy400_0751	MglB>
Chloroflexi	Chloroflexus Y 400 fl	Chy400_2499	<MglB
Chloroflexi	Chloroflexus Y 400 fl	Chy400_3294	<MglB
Chloroflexi	Herpetosiphon aurantiacus DSM 785	Haur_1137	MglB>
Chloroflexi	Herpetosiphon aurantiacus DSM 785	Haur_1268	MglB>
Chloroflexi	Herpetosiphon aurantiacus DSM 785	Haur_1348	<MglB
Chloroflexi	Herpetosiphon aurantiacus DSM 785	Haur_3925	MglB>
Chloroflexi	Herpetosiphon aurantiacus DSM 785	Haur_4223	MglB>
Chloroflexi	Herpetosiphon aurantiacus DSM 785	Haur_4464	MglB>
Chloroflexi	Roseiflexus castenholzii DSM 13941	Rcas_0033	MglB>
Chloroflexi	Roseiflexus castenholzii DSM 13941	Rcas_0335	<MglB
Chloroflexi	Roseiflexus castenholzii DSM 13941	Rcas_1272	<MglB
Chloroflexi	Roseiflexus RS 1	RoseRS_0519	<MglB
Chloroflexi	Roseiflexus RS 1	RoseRS_0746	<MglB
Chloroflexi	Roseiflexus RS 1	RoseRS_0773	<MglB
Crenarchaeota	Caldivirga maquilingensis IC 167	Cmaq_1009	<MglB
Crenarchaeota	Hyperthermus butylicus DSM 5456	Hbut_0175	MglB>
Crenarchaeota	Hyperthermus butylicus DSM 5456	Hbut_1667	<MglB
Crenarchaeota	Ignicoccus hospitalis KIN4 I	Igni_0929	MglB>
Crenarchaeota	Pyrobaculum oguniense TE7	Pogu_1926	<MglB
Crenarchaeota	Pyrolobus fumarii 1A	Pyrfu_1438	<MglB
Crenarchaeota	Pyrolobus fumarii 1A	Pyrfu_1768	MglB>
Crenarchaeota	Vulcanisaeta distributa DSM 14429	Vdis_0327	<MglB
Crenarchaeota	Vulcanisaeta distributa DSM 14429	Vdis_0547	MglB>

Crenarchaeota	Vulcanisaeta moutnovskia 768 28	VMUT_1222	MglB>
Crenarchaeota	Vulcanisaeta moutnovskia 768 28	VMUT_1304	MglB>
Crenarchaeota	Vulcanisaeta moutnovskia 768 28	VMUT_2171	MglB>
Cyanobacteria	Nostoc azollae 0708	Aazo_1029	<MglB
Cyanobacteria	Acaryochloris marina MBIC11017	AM1_1761	MglB>
Cyanobacteria	Acaryochloris marina MBIC11017	AM1_6348	<MglB
Cyanobacteria	Cyanothece ATCC 51142	cce_3011	<MglB
Cyanobacteria	Cyanothece PCC 7424	PCC7424_4094	MglB>
Cyanobacteria	Cyanothece PCC 7424	PCC7424_4311	MglB>
Cyanobacteria	Cyanothece PCC 7822	Cyan7822_0711	<MglB
Cyanobacteria	Cyanothece PCC 7822	Cyan7822_2119	MglB>
Cyanobacteria	Microcystis aeruginosa NIES 843	MAE_52100	MglB>
Deferribacteres	Calditerrivibrio nitroreducens DSM 19672	Calni_0230	MglB>
Deferribacteres	Deferribacter desulfuricans SSM1	DEFDS_0452	MglB>
Deferribacteres	Flexistipes sinusarabici DSM 4947	Flexsi_1825	<MglB
Deinococcus-Thermus	Deinococcus deserti VCD115	Deide_00530	<MglB
Deinococcus-Thermus	Deinococcus deserti VCD115	Deide_12490	<MglB<MglB<MglB
Deinococcus-Thermus	Deinococcus geothermalis DSM 11300	Dgeo_0056	<MglB
Deinococcus-Thermus	Deinococcus geothermalis DSM 11300	Dgeo_1018	MglB>MglB>MglB>
Deinococcus-Thermus	Deinococcus maricopensis DSM 21211	Deima_0656	<MglB
Deinococcus-Thermus	Deinococcus maricopensis DSM 21211	Deima_0832	<MglB<MglB<MglB
Deinococcus-Thermus	Deinococcus proteolyticus MRP	Deipr_0819	MglB>MglB>MglB>
Deinococcus-Thermus	Deinococcus proteolyticus MRP	Deipr_1357	MglB>
Deinococcus-Thermus	Deinococcus proteolyticus MRP	Deipr_1569	<MglB
Deinococcus-Thermus	Deinococcus proteolyticus MRP	Deipr_1709	MglB>
Deinococcus-Thermus	Deinococcus radiodurans R1	DR_0616	MglB>
Deinococcus-Thermus	Deinococcus radiodurans R1	DR_0993	<MglB<MglB<MglB
Deinococcus-Thermus	Deinococcus radiodurans R1	DR_1612	<MglB
Deinococcus-Thermus	Deinococcus radiodurans R1	DR_2347	MglB>
Deinococcus-Thermus	Marinithermus hydrothermalis DSM 14884	Marky_0837	MglB>MglB>MglB>
Deinococcus-Thermus	Meiothermus ruber DSM 1279	Mrub_1151	<MglB<MglB
Deinococcus-Thermus	Meiothermus silvanus DSM 9946	Mesil_1376	<MglB<MglB
Deinococcus-Thermus	Oceanithermus profundus DSM 14977	Ocepr_1372	<MglB<MglB<MglB
Deinococcus-Thermus	Thermus scotoductus SA 01	TSC_c21340	MglB>MglB>MglB>
Deinococcus-Thermus	Thermus thermophilus HB27	TTC1237	MglB>MglB>MglB>
Deinococcus-Thermus	Thermus thermophilus HB8	TTHA1601	MglB>MglB>MglB>
Deinococcus-Thermus	Truepera radiovictrix DSM 17093	Trad_1688	<MglB
Dictyoglomi	Dictyoglomus thermophilum H 6 12	DICTH_1004	MglB>
Dictyoglomi	Dictyoglomus turgidum DSM 6724	Dtur_1132	MglB>
Euryarchaeota	Aciduliprofundum boonei T469	Aboo_0096	<MglB
Euryarchaeota	Aciduliprofundum boonei T469	Aboo_0216	MglB>
Euryarchaeota	Archaeoglobus fulgidus DSM 4304	AF1500	MglB>
Euryarchaeota	Archaeoglobus veneficus SNP6	Arcve_1632	MglB>
Euryarchaeota	Methanobacterium AL 21	Metbo_0394	<MglB
Euryarchaeota	Methanobacterium AL 21	Metbo_0638	<MglB
Euryarchaeota	Methanobacterium AL 21	Metbo_0652	<MglB
Euryarchaeota	Methanobacterium AL 21	Metbo_1335	MglB>
Euryarchaeota	Methanobacterium SWAN 1	MSWAN_1706	MglB>
Euryarchaeota	Methanobacterium SWAN 1	MSWAN_1718	MglB>
Euryarchaeota	Methanocaldococcus fervens AG86	Mefer_0193	MglB>
Euryarchaeota	Methanocaldococcus fervens AG86	Mefer_0869	<MglB
Euryarchaeota	Methanocaldococcus FS406 22	MFS40622_0110	MglB>
Euryarchaeota	Methanocaldococcus FS406 22	MFS40622_0465	<MglB
Euryarchaeota	Methanocaldococcus FS406 22	MFS40622_0920	<MglB
Euryarchaeota	Methanocaldococcus FS406 22	MFS40622_0951	MglB>
Euryarchaeota	Methanocaldococcus FS406 22	MFS40622_1083	<MglB<MglB
Euryarchaeota	Methanocaldococcus FS406 22	MFS40622_1457	<MglB
Euryarchaeota	Methanocaldococcus infernus ME	Metin_0518	<MglB
Euryarchaeota	Methanocaldococcus infernus ME	Metin_1043	<MglB

Euryarchaeota	Methanocaldococcus infernus ME	Metin_1123	<MglB
Euryarchaeota	Methanocaldococcus jannaschii DSM 2661	MJ_0163	<MglB
Euryarchaeota	Methanocaldococcus jannaschii DSM 2661	MJ_0310	<MglB<MglB
Euryarchaeota	Methanocaldococcus jannaschii DSM 2661	MJ_0714	MglB>
Euryarchaeota	Methanocaldococcus jannaschii DSM 2661	MJ_1389	<MglB
Euryarchaeota	Methanocaldococcus vulcanius M7	Metvu_0503	MglB>
Euryarchaeota	Methanocaldococcus vulcanius M7	Metvu_1411	MglB>
Euryarchaeota	Methanocaldococcus vulcanius M7	Metvu_1655	<MglB
Euryarchaeota	Methanocella paludicola SANAE	MCP_0104	<MglB
Euryarchaeota	Methanocella paludicola SANAE	MCP_0845	MglB>
Euryarchaeota	Methanocella paludicola SANAE	MCP_2490	<MglB<MglB
Euryarchaeota	Methanocella paludicola SANAE	MCP_2963	<MglB
Euryarchaeota	Methanococcus aeolicus Nankai 3	Maeo_0695	MglB>
Euryarchaeota	Methanococcus aeolicus Nankai 3	Maeo_0731	<MglB
Euryarchaeota	Methanococcus maripaludis C5	MmarC5_0812	MglB>
Euryarchaeota	Methanococcus maripaludis C6	MmarC6_1665	<MglB
Euryarchaeota	Methanococcus maripaludis C7	MmarC7_0234	MglB>
Euryarchaeota	Methanococcus maripaludis S2	MMP0992	MglB>
Euryarchaeota	Methanococcus maripaludis X1	GYY_05790	MglB>
Euryarchaeota	Methanococcus vannielii SB	Mevan_0319	MglB>
Euryarchaeota	Methanoculleus marisnigri JR1	Memar_0294	MglB>
Euryarchaeota	Methanoculleus marisnigri JR1	Memar_0308	MglB>
Euryarchaeota	Methanoculleus marisnigri JR1	Memar_1005	<MglB<MglB
Euryarchaeota	Methanoculleus marisnigri JR1	Memar_1260	MglB>
Euryarchaeota	Methanoplanus petrolearius DSM 11571	Mpet_1740	<MglB
Euryarchaeota	Methanoplanus petrolearius DSM 11571	Mpet_2716	<MglB<MglB
Euryarchaeota	Methanopyrus kandleri AV19	MK0216	MglB>
Euryarchaeota	Methanopyrus kandleri AV19	MK1325	MglB>
Euryarchaeota	Methanoregula boonei 6A8	Mboo_1907	<MglB<MglB
Euryarchaeota	Methanoregula boonei 6A8	Mboo_1938	<MglB
Euryarchaeota	Methanosphaerula palustris E1 9c	Mpal_2155	MglB>
Euryarchaeota	Methanospirillum hungatei JF 1	Mhun_1617	MglB>
Euryarchaeota	Methanothermobacter marburgensis Marburg	MTBMA_c04060	<MglB
Euryarchaeota	Methanothermobacter marburgensis Marburg	MTBMA_c08940	<MglB
Euryarchaeota	Methanothermobacter marburgensis Marburg	MTBMA_c09750	<MglB
Euryarchaeota	Methanothermobacter thermautotrophicus Delta H	MTH503	<MglB
Euryarchaeota	Methanothermobacter thermautotrophicus Delta H	MTH595	<MglB
Euryarchaeota	Methanothermobacter thermautotrophicus Delta H	MTH1836	<MglB
Euryarchaeota	Methanothermococcus okinawensis IH1	Metok_0392	<MglB
Euryarchaeota	Methanothermus fervidus DSM 2088	Mfer_0303	<MglB
Euryarchaeota	Methanothermus fervidus DSM 2088	Mfer_0948	<MglB
Euryarchaeota	Methanotorris igneus Kol 5	Metig_0414	<MglB
Euryarchaeota	Thermococcus AM4	TAM4_1964	<MglB
Euryarchaeota	Thermococcus gammatolerans EJ3	TGAM_1884	<MglB
Euryarchaeota	uncultured methanogenic archaeon RC I	RCIX930	MglB>
Euryarchaeota	uncultured methanogenic archaeon RC I	RCIX472	MglB>MglB>
Euryarchaeota	uncultured methanogenic archaeon RC I	RCIX296	<MglB
Fibrobacteres	Fibrobacter succinogenes S85	Fisuc_0226	<MglB
Gemmatimonadetes	Gemmatimonas aurantiaca T 27	GAU_1541	<MglB
Proteobacteria-alpha	Bradyrhizobium BTAi1	BBta_6136	MglB>
Proteobacteria-alpha	Bradyrhizobium ORS 278	BRADO5612	MglB>
Proteobacteria-beta	Leptothrix cholodnii SP 6	Lcho_2108	MglB>
Proteobacteria-beta	Neisseria gonorrhoeae FA 1090	NGO1475	<MglB<MglB
Proteobacteria-beta	Neisseria gonorrhoeae NCCP11945	NGK_1742	<MglB<MglB
Proteobacteria-beta	Polaromonas naphthalenivorans CJ2	Pnap_2813	<MglB
Proteobacteria-beta	Ramlibacter tataouinensis TTB310	Rta_26070	<MglB
Proteobacteria-delta	Anaeromyxobacter dehalogenans 2CP 1	A2cp1_0681	MglB>
Proteobacteria-delta	Anaeromyxobacter dehalogenans 2CP C	Adeh_0647	MglB>
Proteobacteria-delta	Anaeromyxobacter Fw109 5	Anae109_0692	MglB>

Proteobacteria-delta	Anaeromyxobacter K	AnaeK_0681	MglB>
Proteobacteria-delta	Corallococcus coralloides DSM 2259	COCOR_06270	<MglB
Proteobacteria-delta	Desulfatibacillum alkenivorans AK 01	Dalk_2359	MglB>
Proteobacteria-delta	Geobacter bemidjiensis Bem	Gbem_0963	MglB>
Proteobacteria-delta	Geobacter bemidjiensis Bem	Gbem_1558	MglB>
Proteobacteria-delta	Geobacter FRC 32	Geob_3060	<MglB
Proteobacteria-delta	Geobacter FRC 32	Geob_3596	<MglB
Proteobacteria-delta	Geobacter lovleyi SZ	Glov_0343	<MglB
Proteobacteria-delta	Geobacter lovleyi SZ	Glov_1594	MglB>
Proteobacteria-delta	Geobacter lovleyi SZ	Glov_3495	MglB>
Proteobacteria-delta	Geobacter M18	GM18_0862	MglB>
Proteobacteria-delta	Geobacter M18	GM18_1393	MglB>
Proteobacteria-delta	Geobacter M21	GM21_2658	<MglB
Proteobacteria-delta	Geobacter M21	GM21_3298	<MglB
Proteobacteria-delta	Geobacter metallireducens GS 15	Gmet_0829	MglB>
Proteobacteria-delta	Geobacter metallireducens GS 15	Gmet_0980	MglB>
Proteobacteria-delta	Geobacter metallireducens GS 15	Gmet_1175	MglB>
Proteobacteria-delta	Geobacter sulfurreducens PCA	GSU0316	<MglB
Proteobacteria-delta	Geobacter sulfurreducens PCA	GSU1131	MglB>
Proteobacteria-delta	Geobacter sulfurreducens PCA	GSU2023	<MglB
Proteobacteria-delta	Geobacter sulfurreducens PCA	GSU2641	<MglB
Proteobacteria-delta	Geobacter uraniireducens Rf4	Gura_1095	MglB>
Proteobacteria-delta	Geobacter uraniireducens Rf4	Gura_1822	MglB>
Proteobacteria-delta	Geobacter uraniireducens Rf4	Gura_2951	MglB>
Proteobacteria-delta	Geobacter uraniireducens Rf4	Gura_3343	<MglB
Proteobacteria-delta	Haliangium ochraceum DSM 14365	Hoch_0254	MglB>
Proteobacteria-delta	Haliangium ochraceum DSM 14365	Hoch_2376	<MglB
Proteobacteria-delta	Hippea maritima DSM 10411	Hipma_0913	<MglB
Proteobacteria-delta	Myxococcus fulvus HW 1	LILAB_36340	<MglB
Proteobacteria-delta	Myxococcus xanthus DK 1622	MXAN_5770	<MglB
Proteobacteria-delta	Pelobacter carbinolicus DSM 2380	Pcar_2130	<MglB
Proteobacteria-delta	Pelobacter carbinolicus DSM 2380	Pcar_2355	<MglB
Proteobacteria-delta	Pelobacter carbinolicus DSM 2380	Pcar_2475	MglB>
Proteobacteria-delta	Pelobacter propionicus DSM 2379	Ppro_2025	<MglB
Proteobacteria-delta	Pelobacter propionicus DSM 2379	Ppro_3142	MglB>
Proteobacteria-delta	Pelobacter propionicus DSM 2379	Ppro_3318	<MglB
Proteobacteria-delta	Sorangium cellulosum So ce 56	sce2584	MglB>
Proteobacteria-delta	Sorangium cellulosum So ce 56	sce3925	<MglB
Proteobacteria-delta	Stigmatella aurantiaca DW4 3 1	STAUR_6439	<MglB
Proteobacteria-epsilon	Sulfurovum NBC37 1	SUN_1399	<MglB
Proteobacteria-gamma	Acidithiobacillus caldus SM 1	Atc_1690	MglB>
Proteobacteria-gamma	Acidithiobacillus ferrivorans SS3	Acife_0732	MglB>---MglB>
Proteobacteria-gamma	Acidithiobacillus ferrivorans SS3	Acife_1074	MglB>
Proteobacteria-gamma	Acidithiobacillus ferrooxidans ATCC 23270	AFE_0401	<MglB
Proteobacteria-gamma	Acidithiobacillus ferrooxidans ATCC 23270	AFE_0978	MglB>
Proteobacteria-gamma	Acidithiobacillus ferrooxidans ATCC 23270	AFE_2255	<MglB
Proteobacteria-gamma	Acidithiobacillus ferrooxidans ATCC 53993	Lferr_0565	<MglB
Proteobacteria-gamma	Acidithiobacillus ferrooxidans ATCC 53993	Lferr_1092	MglB>
Proteobacteria-gamma	Acidithiobacillus ferrooxidans ATCC 53993	Lferr_1902	<MglB
Proteobacteria-gamma	Methylomonas methanica MC09	Metme_2245	<MglB<MglB
Proteobacteria-gamma	Nitrosococcus halophilus Nc4	Nhal_2891	<MglB
Proteobacteria-gamma	Nitrosococcus oceani ATCC 19707	Noc_1488	<MglB
Proteobacteria-gamma	Nitrosococcus watsonii C 113	Nwat_1624	MglB>
Proteobacteria-gamma	Pseudoxanthomonas suwonensis 11 1	Psesu_2129	MglB>
Proteobacteria-gamma	Xanthomonas campestris vesicatoria 85 10	XCV4103	MglB>
Proteobacteria-gamma	Xanthomonas oryzae KACC10331	XOO0434	<MglB
Proteobacteria-gamma	Xanthomonas oryzae MAFF 311018	XOO_0396	<MglB
Proteobacteria-gamma	Xanthomonas oryzae PXO99A	PXO_02849	MglB>
Verrucomicrobia	Methylacidiphilum infernorum V4	Minf_1089	<MglB

Table S4. Domain architecture analysis of 134 MglA (MXAN_1925) BLAST hits with an e-value of 0.0001 or better.

Pfam domain	HMM threshold[1]	Top HMM[2]
Arf	109	75
ATP_bind_1	19	13
Dynamin_N	1	0
FeoB_N	3	0
GTP_EFTU	29	1
Gtr1_RagA	21	3
Miro	48	15
MMR_HSR1	4	0
Ras	55	12
SRPRB	9	0
None	15	15

[1] HMM threshold indicates the number of BLAST hits with sequences that match the corresponding Pfam domain model within the gathering threshold unless no domains are identified (None).

[2] Top HMM indicates the number of BLAST hits with sequences that have the corresponding Pfam domain as the top-scoring model when multiple match models within the gathering threshold overlap.
