# Supplementary material for: Evolution and Diversity of the Ras Superfamily of Small GTPases in Prokaryotes
Source: Genome Biol Evol. 2014 Dec 4;7(1):57–70. doi: 10.1093/gbe/evu264 (PMC4316618; doi:10.1093/gbe/evu264)
Supplement: Supplementary Data [file supp_evu264_New_Microsoft_Office_Word_Document.docx]

**Supplementary Material**

**Figure S1.** Structure of MglA-MglB complex of *Thermus thermophilus*.

**Figure S2.** Histidine kinases (HKs) encoded with the Group 2 conservon form a distinct subfamily.

**Table S1.** Domain architecture analysis of 63 MglB (MXAN_1926) BLAST hits with an e-value of 0.0001 or better.

**Table S2.** Identified MglA and Rup GTPases as well as coupled MglB sequences.

**Table S3.** Orphan MglB sequences.

**Table S4.** Domain architecture analysis of 134 MglA (MXAN_1925) BLAST hits with an evalue of 0.0001 or better
